# Hyaluronic acid-based tissue-engineered biomaterials for infected diabetic foot ulcers: mechanisms, evidence, and translational challenges

**DOI:** 10.3389/fbioe.2026.1963864

**Published:** 2026-09-22

**Authors:** Chenyu Wang, Zuofa Yan, Zeyang Zhang, Di Wu, Xinli Dong, Yujin Dong

**Affiliations:** Department of Orthopedics, Affiliated Zhongshan Hospital of Dalian University, Dalian, China

**Keywords:** biofilm, diabetic foot ulcer, hyaluronic acid, hydrogel, infected chronic wound, pathological microenvironment, tissue-engineered biomaterial

## Abstract

Diabetic foot ulcers (DFUs) have difficulty healing clinically when infection and mature biofilms coexist with hyperglycemic injury, immune dysregulation, oxidative stress, ischemia–hypoxia, repetitive mechanical loading, and extracellular matrix (ECM) degradation. Debridement, infection management, offloading, vascular assessment and revascularization, when indicated, constitute the foundation of care; biomaterials can be evaluated only as adjuncts to this pathway. Hyaluronic acid (HA) is an attractive hydrated ECM-associated and chemically adaptable matrix that can support local retention, controlled release and cell‒material interactions. It is not intrinsically superior to chitosan, alginate, collagen/gelatin or synthetic polymers in every respect: unmodified HA has limited antimicrobial activity and insufficient mechanical strength for many plantar applications. Moreover, antimicrobial, antioxidant, immunomodulatory and angiogenic effects in multicomponent systems are often provided primarily by incorporated antibiotics, metal ions, polyphenols, nitric oxide donors, growth factors or extracellular vesicles rather than by HA itself. This Review therefore distinguishes the contribution of the HA matrix from that of active cargo and separates direct evidence from explicit infected diabetic wound models, indirect evidence from noninfected diabetic wounds, *in vitro* mechanistic evidence and clinical DFU studies in which infection status was not reported or stratified. Only a minority of preclinical studies have used explicit infected diabetic wound models, and almost all published HA clinical studies have included general populations of DFU patients without biofilm or perfusion stratification. The proposed matching of HA platforms to biofilm-, inflammation/ROS-, ischemia- or ECM-dominant phenotypes is therefore a testable translational hypothesis, not a guideline-endorsed treatment algorithm. Progress requires claim-matched models, full reporting of the HA source, molecular weight distribution, and purification method, endotoxin burden, component deconvolution controls, stage-responsive rather than indiscriminate multifunctionality, long-term local and systemic safety monitoring, and clinical outcomes extending beyond short-term wound closure.

## Graphical Abstract

**Figure:**
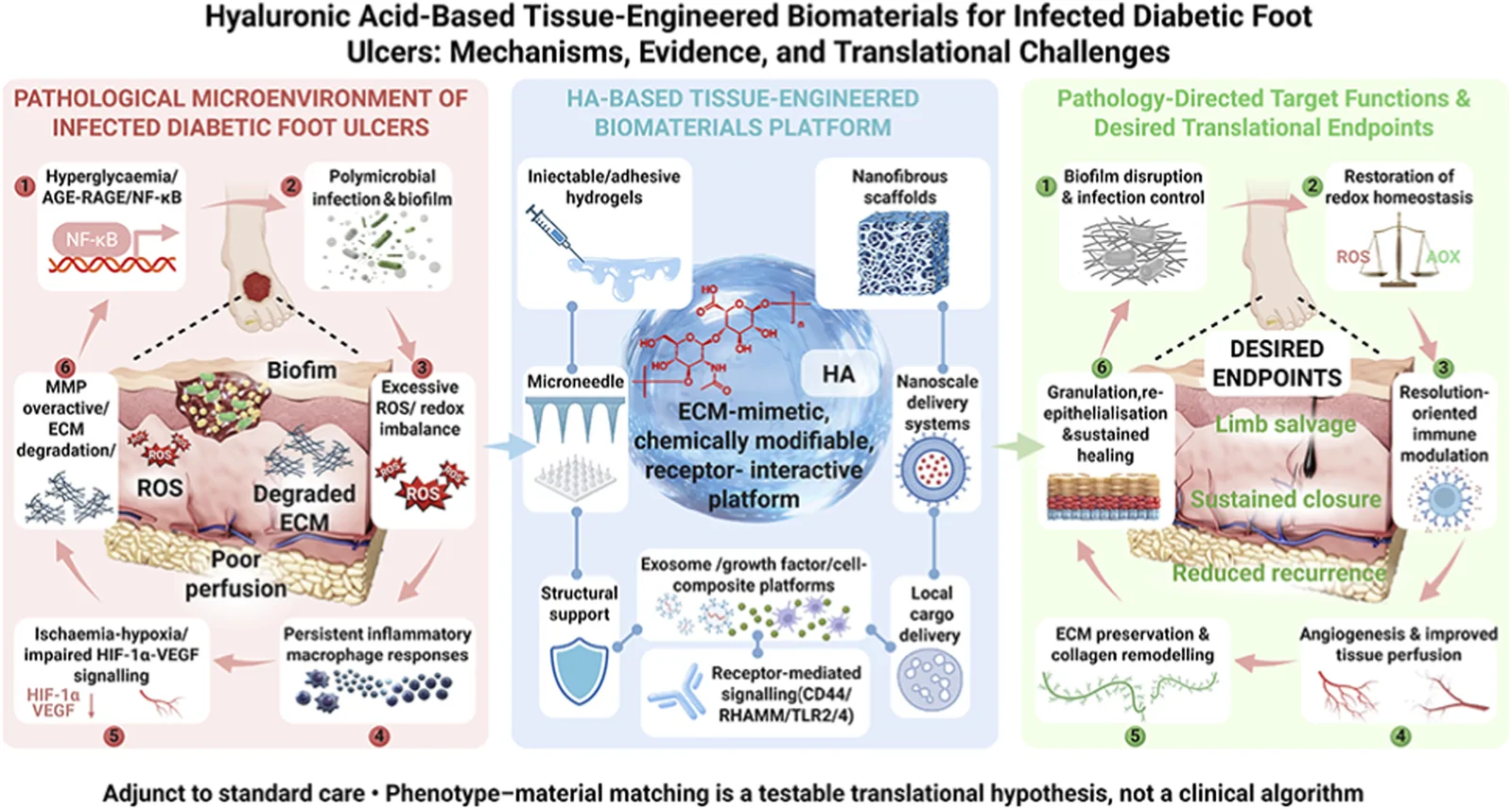
HA-based platforms are presented as pathology-directed adjuncts. Limb salvage, sustained wound closure and reduced recurrence are desired translational endpoints, not outcomes already established with HA-based materials.

## 1 Introduction

Diabetic foot ulcers (DFUs) are among the most consequential chronic complications of diabetes and are associated with recurrent ulceration, infection, hospitalization, lower extremity amputation and reduced long-term survival (Armstrong et al., 2023; McDermott et al., 2023). Their development reflects the combined effects of hyperglycemia, peripheral neuropathy, peripheral arterial disease (PAD), abnormal plantar loading, impaired host defense and defective repair (Wang et al., 2022; Edmonds et al., 2021). Once infection develops, deep tissue extension, osteomyelitis and limb loss become more likely, while apparent wound closure does not eliminate the risk of recurrence. Infected DFUs should therefore be treated as complex, recurrent lesions rather than isolated cutaneous defects.

Infection changes the problem for which wound biomaterials are designed. In addition to bacterial burden, infected DFUs may contain mature polymicrobial biofilms with antimicrobial tolerance, sustained innate immune activation and microbial proteases. Hyperglycemia amplifies AGE-RAGE/NF-κB signaling; an excess of reactive oxygen species (ROS) impairs fibroblasts, keratinocytes and endothelial cells; ischemia restricts oxygen and antimicrobial delivery; and excessive matrix metalloproteinase (MMP) activity degrades extracellular matrix (ECM) proteins and growth factors (Burgess et al., 2021; Mohsin et al., 2024; Theodorakopoulos and Armstrong, 2025). These abnormalities reinforce one another, so killing the causative agent alone is unlikely to restore durable repair.

This biological complexity does not reduce the primacy of standard DFU care. Debridement and drainage, infection grading and antimicrobial treatment, offloading, vascular assessment and revascularization when indicated, metabolic management and multidisciplinary follow-up remain indispensable (Chen et al., 2024; Bus et al., 2024; Senneville et al., 2024; Fitridge et al., 2024). Any HA-based material must be tested as an adjunct to fully delivered standard care, not as an alternative to source control, pressure relief or the restoration of perfusion.

HA is prioritized here because it combines four properties within one platform: it is an ECM-associated, highly hydrated polymer; its carboxyl and hydroxyl groups are easily modified; it forms degradable matrices capable of retaining labile cargo; and it interacts with receptors such as CD44 and RHAMM. These features can support wound conformity, cell migration, local delivery and material responsiveness (Bokatyi et al., 2024; Chang et al., 2024; Yang et al., 2021). The choice of biomaterial remains conditional. Chitosan offers stronger intrinsic cationic antimicrobial activity, alginate efficiently manages exudate, collagen/gelatin supplies cell-adhesive motifs, and PEG/GelMA may provide more predictable mechanical properties. Unmodified HA has limited intrinsic antimicrobial activity, is rapidly degraded and is mechanically weak; thus, it must often be crosslinked or combined with other polymers (Valachová et al., 2022; Xiang et al., 2024).

A second distinction is essential. In composite systems, HA may form the matrix, improve injectability or adhesion, control degradation, retain cargo and influence receptor-associated cell behavior. In contrast, antibiotics, metal ions, photothermal agents, polyphenols, nitric oxide donors, growth factors or extracellular vesicles frequently provide the dominant antimicrobial, antioxidant, immunomodulatory or angiogenic effects. The therapeutic efficacy should not be attributed to HA unless component deconvolution controls demonstrate an independent contribution.

Most recent reviews have organized HA materials according to hydrogel chemistry, crosslinking, self-healing behavior, stimulus-responsive release or drug delivery format (Chhillar and Jaiswal, 2025; Liu et al., 2025; Zhang et al., 2025), whereas the present Review adds an infection-specific and translational lens. It links clinical phenotype and material constraints to HA biology; distinguishes direct infection model evidence from indirect evidence; separates matrix and cargo contributions; and evaluates model relevance, manufacturing, safety and clinically meaningful outcomes. The proposed phenotype–material matching framework is explicitly hypothesis-generating and requires prospective validation.

## 2 Clinical phenotypes and material design constraints

The clinical problem is not simply whether a material accelerates wound closure but whether it remains usable within a pathway that includes repeated assessment, debridement, dressing changes and the escalation of infection or vascular care. A platform that obstructs drainage, masks deterioration, cannot be removed atraumatically or fails under loading may have little clinical value even when it improves a dorsal rodent wound histologically. Material selection and study eligibility should therefore be anchored to the wound phenotype and practical treatment pathway.

For biomaterial development, the clinically relevant variables are those that alter indication, material performance or interpretation of efficacy, including infection severity, perfusion, wound depth, necrosis, exudate, suspected osteomyelitis, neuropathy and mechanical loading. Classification systems such as SINBAD, WIfI and the IWGDF/IDSA infection grade can standardize baseline descriptions and stratification (Monteiro-Soares et al., 2024; Mills et al., 2014; Senneville et al., 2024). A locally applied material cannot be interpreted independently of these variables.

### 2.1 Infection, perfusion, wound geometry and exudate

Clinical infection is diagnosed on the basis of local or systemic inflammatory findings, not culture positivity alone, and colonization does not establish diabetic foot infection (Senneville et al., 2024). Antimicrobial HA platforms should therefore be evaluated against viable tissue burden and the presence of a mature biofilm with infection resolution, repeated debridement and tissue repair as outcomes. Inhibition zones and planktonic colony counts are preliminary tests rather than clinical surrogates (Chen et al., 2022; Theodorakopoulos and Armstrong, 2025).

Perfusion defines whether local repair is biologically plausible. PAD assessment requires clinical examination and appropriate physiological testing, whereas severe ischemia requires vascular management rather than a proangiogenic dressing alone (Fitridge et al., 2024). Wound depth, sinus tracts, dead space, necrosis and exudate likewise determine whether an injectable, adhesive, fibrous or microneedle format is suitable. Injectable hydrogels have been proposed as an option for selected irregular postdebridement defects, but deep infection immediately increases the need for source control, drainage, viable tissue and repeated access. This is an engineering hypothesis, not direct clinical evidence for filling infected cavities.

### 2.2 Mechanical loading and the nonnegotiable foundation of standard care

Offloading is central to the healing of neuropathic plantar ulcers (Bus et al., 2024). Materials intended for the plantar surface must therefore be evaluated for adhesion, recovery after deformation, fatigue, shear stability and performance during prolonged fluid exposure. Conventional streptozotocin-induced or db/db mouse dorsal wounds cannot reliably assess responses to plantar weight-bearing, wound exudate dynamics under loading, adhesive hydrogel retention under cyclic shear, or recurrence after a return to activity (Hu et al., 2022; Du et al., 2023; Couturier et al., 2023). Wound closure in these models supports preliminary repair activity, not plantar mechanical competence. Recent systematic, in-shoe and gait evidence confirms that plantar shear, in addition to pressure, is measurable and clinically relevant (Jones et al., 2022; Tang et al., 2023; Mima et al., 2025).

Debridement controls devitalized tissue and improves material contact with the wound bed; antimicrobial treatment can be used to treat clinically defined infections; offloading can reduce repetitive injury; and revascularization can be used to address major limitations of perfusion. HA materials may prolong local exposure, provide a temporary matrix or modulate residual biological barriers after these measures have been administered (Dayya et al., 2022; Chen et al., 2024). Trials must document debridement, antimicrobial regimens, offloading adherence and vascular interventions so that the independent added effect of the material can be estimated.

A systematic review, meta-analysis and meta-regression of 30 studies revealed only weak evidence that any debridement method was superior to another method or control; consequently, trials of HA materials should report debridement modality and frequency rather than treat ‘standard care’ as a uniform comparator (Dayya et al., 2022).

### 2.3 Phenotype–material matching: a testable translational hypothesis

Biofilm-dominant wounds may motivate antimicrobial or local delivery platforms; inflammation/ROS-dominant wounds may motivate redox-responsive or immunomodulatory systems; ischemia-dominant wounds may motivate angiogenesis-supporting material delivery after vascular optimization; and ECM degradation-dominant wounds may motivate ECM-mimetic or MMP-responsive matrices. This framework is proposed for material design and study stratification. It is not a guideline-endorsed clinical algorithm and must not be applied before debridement, offloading, infection management and vascular optimization have been fully addressed (Schaper et al., 2024; Zhao et al., 2024).

## 3 The pathological microenvironment as a set of material design targets

The principal pathological axes are summarized in Figure 1. Complementary schematics illustrating macrophage-associated inflammation, angiogenesis, HA modification and RHAMM signaling, together with representative responsive hydrogels, nanofibers, microneedles, nanomedicines, extracellular-vesicle-mediated repair, and multifunctional composite systems, are presented in Figures 2–11. They are presented as linked design targets rather than independent mechanisms: hyperglycemia amplifies inflammation and ROS production; infection and biofilms sustain immune activation; ischemia limits host defense and repair; and ROS, inflammatory mediators and microbial proteases accelerate ECM damage.

**FIGURE 1:**
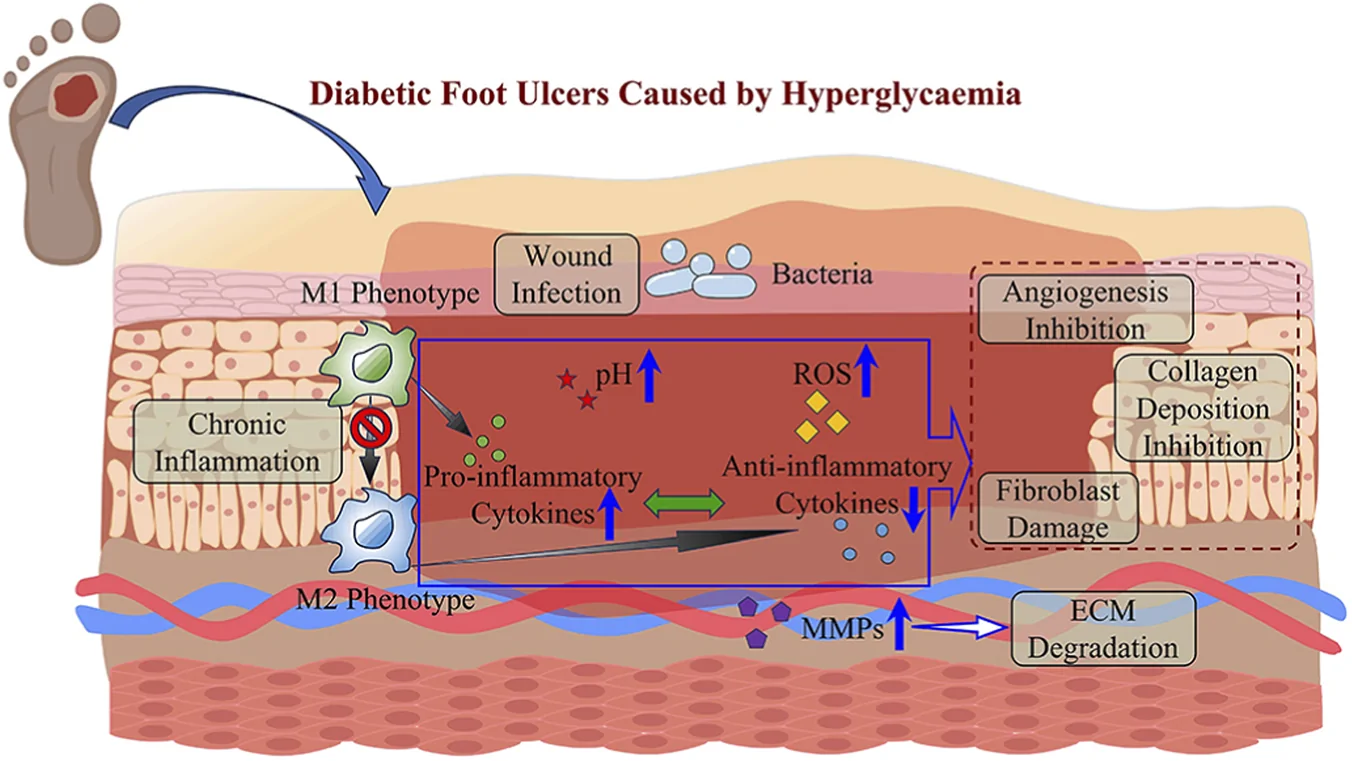
Pathological microenvironment of diabetic wounds. Hyperglycemic injury, bacterial infection, excessive ROS, persistent inflammatory macrophage responses, impaired angiogenesis, fibroblast dysfunction, excessive MMP activity and ECM degradation contribute to delayed repair. The source figure depicts diabetic wound biology and should not be interpreted as direct evidence from an infected plantar DFU model. Reproduced from Zhang et al. (2025a), with permission from Elsevier.

**FIGURE 2:**
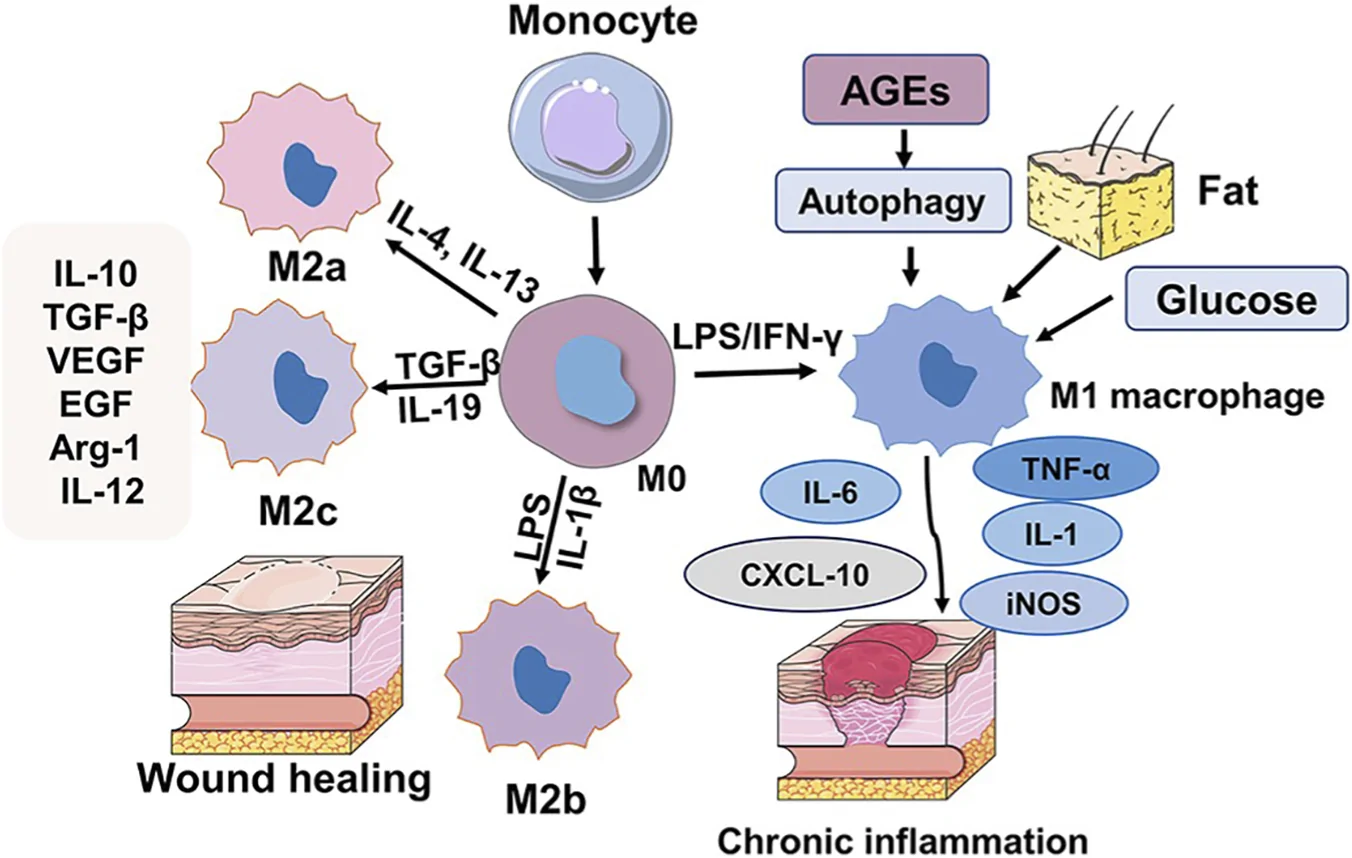
Simplified representation of macrophage-associated inflammation and repair functions during the healing of diabetic wounds. M1/M2 labels are heuristic and do not capture the full temporal and functional heterogeneity of macrophages in wounds. Reproduced from Wu et al. (2022) under the Creative Commons Attribution 4.0 International License.

**FIGURE 3:**
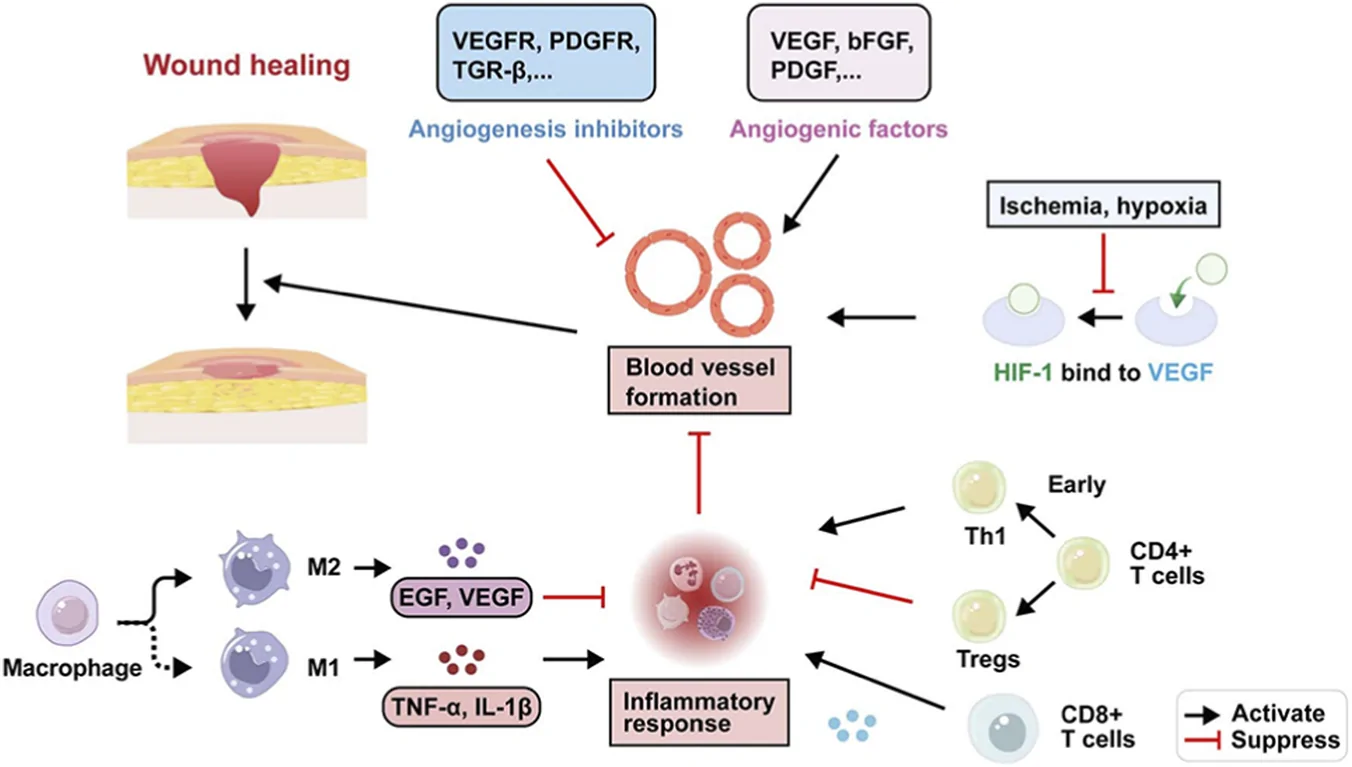
Cellular and molecular events in angiogenesis during wound repair. Endothelial activation, matrix degradation, migration, sprouting, lumen formation and vessel maturation must be coordinated. Reproduced from Huang et al. (2025) under the Creative Commons Attribution 4.0 International License.

**FIGURE 4:**
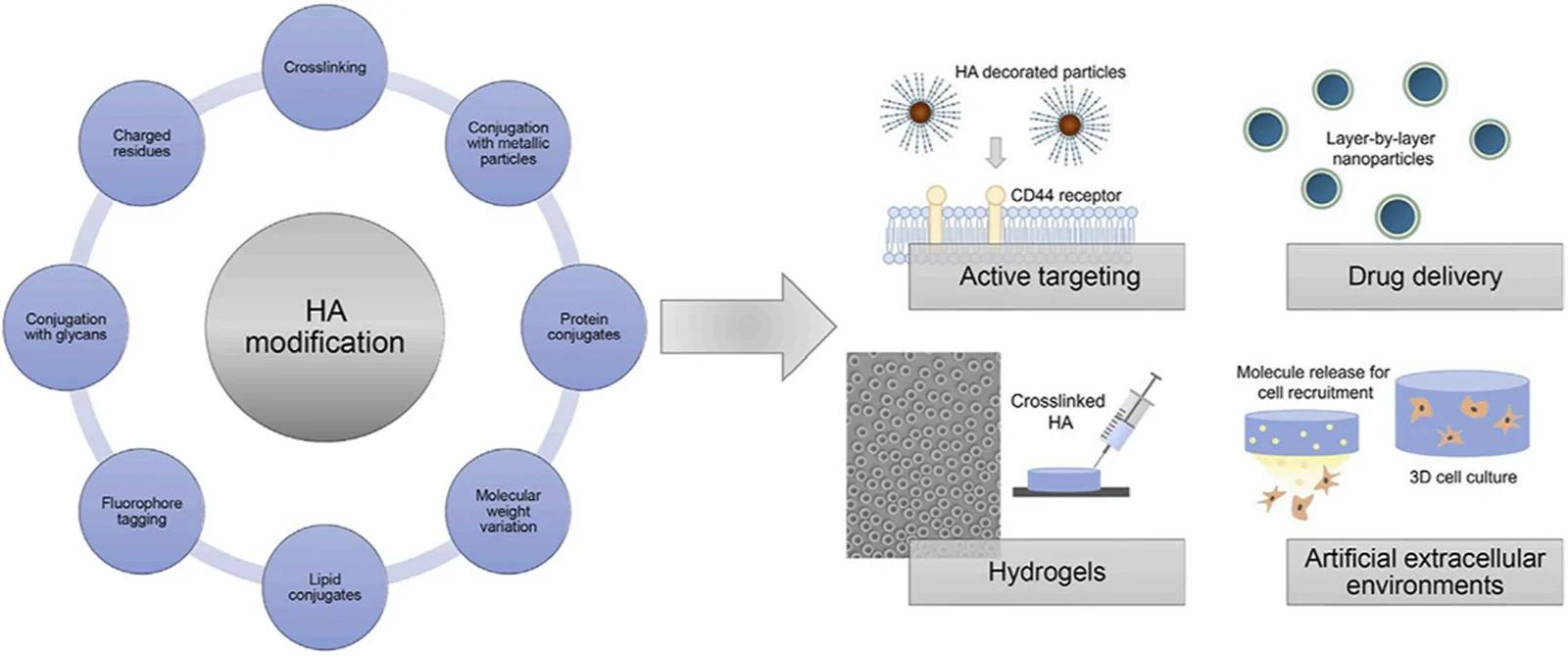
Major chemical modification strategies for HA and their biomedical applications. Reproduced from Hintze et al. (2022) under the Creative Commons Attribution 4.0 International License.

**FIGURE 5:**
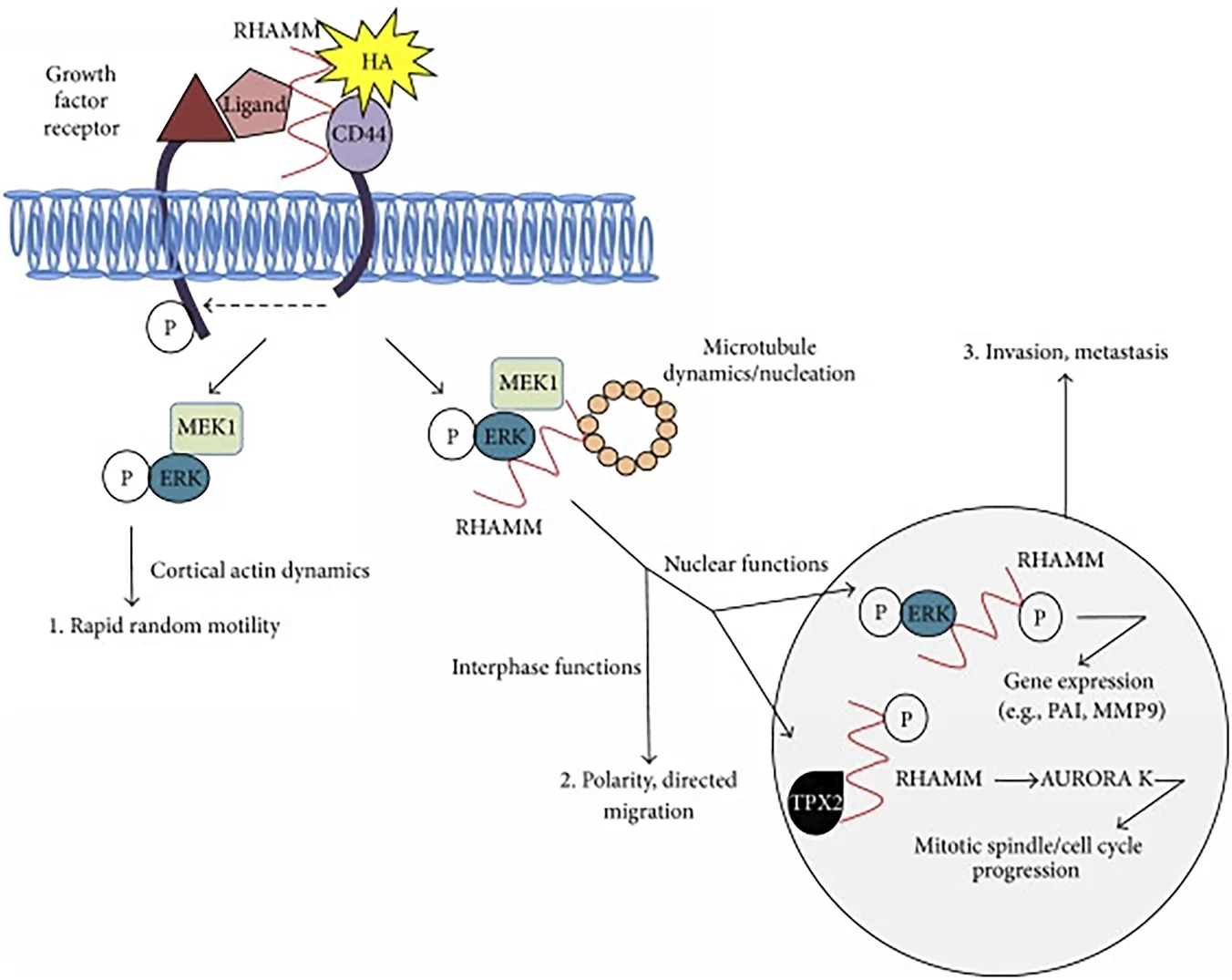
Extracellular and intracellular RHAMM signaling is important during wound repair. Reproduced without modification from Tolg et al. (2014) under the Creative Commons Attribution License.

**FIGURE 6:**
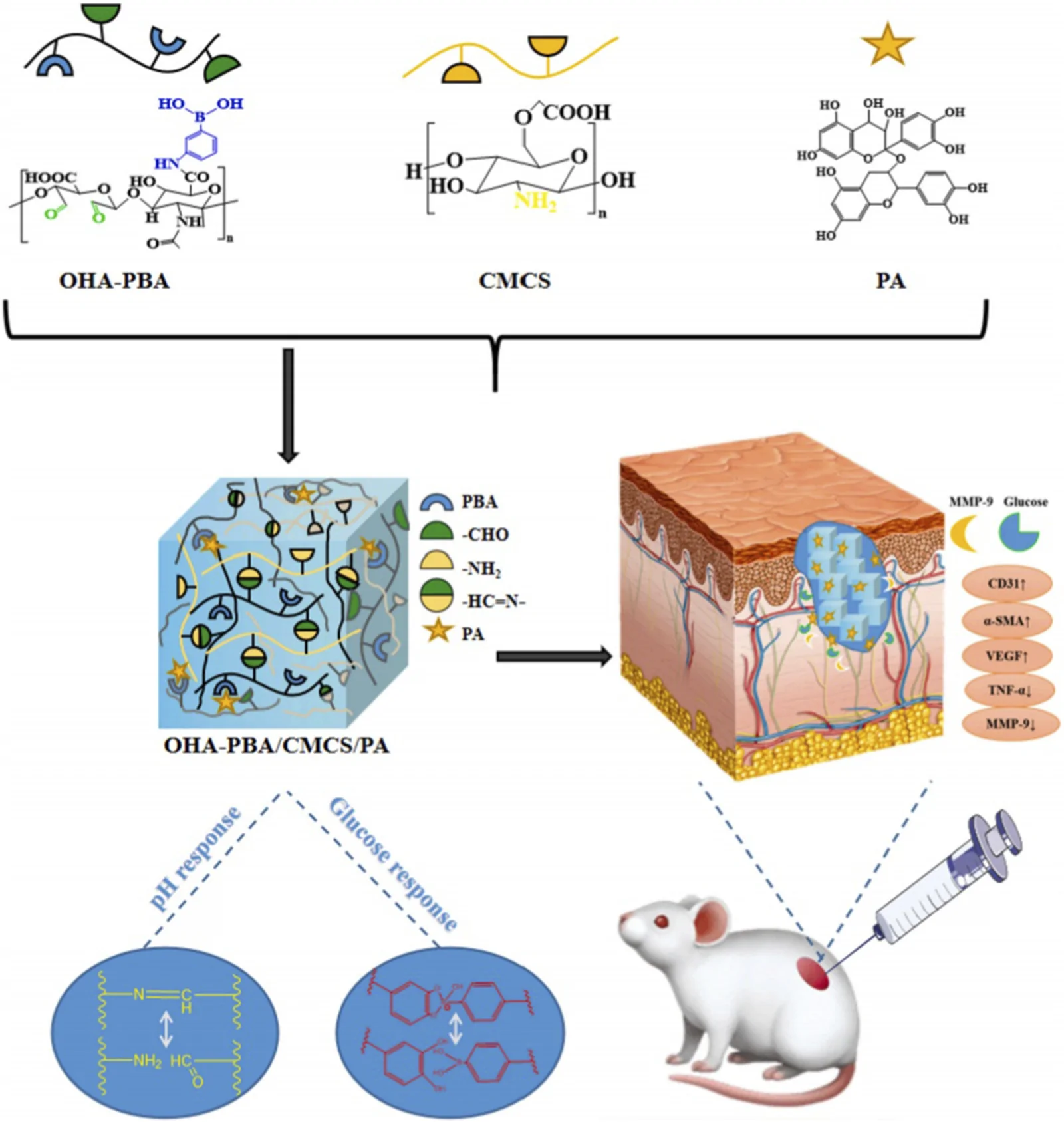
Construction and release mechanism of a pH- and glucose-responsive HA-containing antioxidant hydrogel. The reported repair outcomes were obtained in a noninfected experimental diabetic wound model and should be interpreted as indirect evidence for the treatment of infected DFUs. Reproduced from Zhang et al. (2025b) with permission from Elsevier.

**FIGURE 7:**
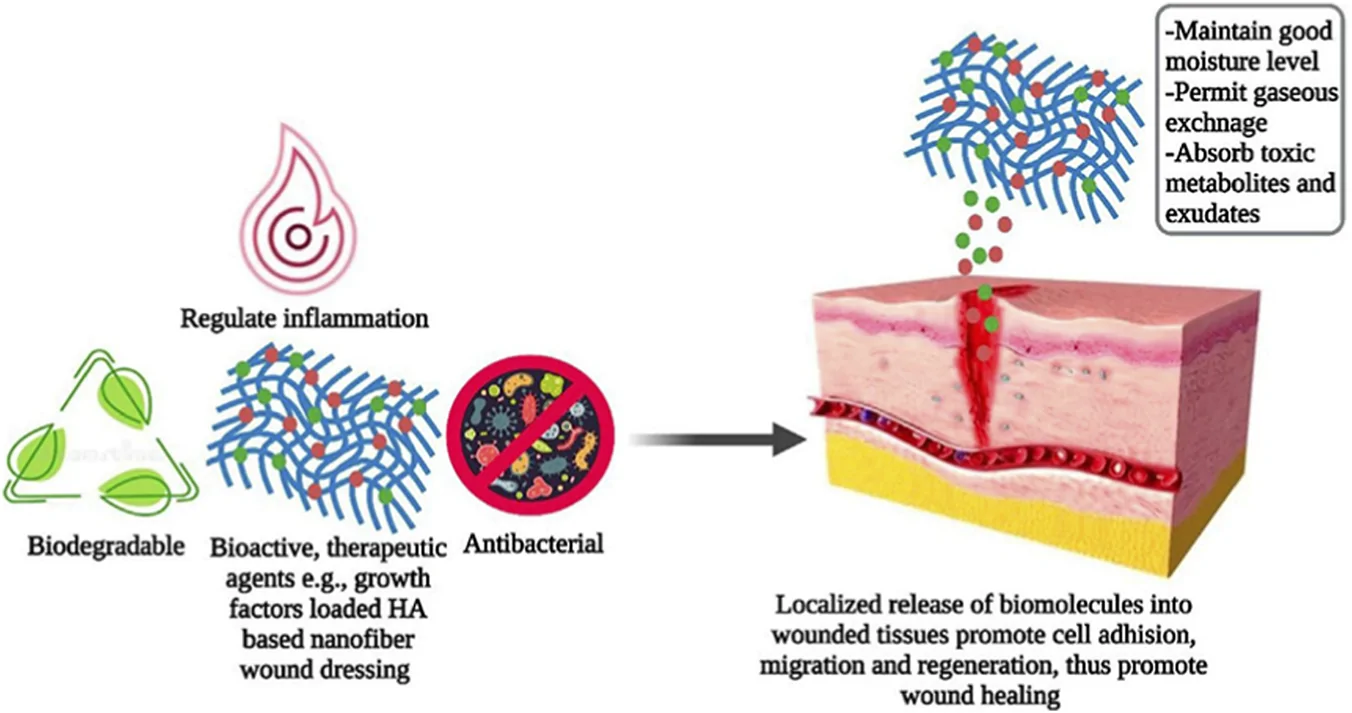
Mechanisms by which HA-based nanofibrous dressings may support wound repair, including ECM mimicry, local drug delivery and cell migration. This is a platform schematic rather than direct evidence of DFU infection. Reproduced from Humaira et al. (2022) under the Creative Commons Attribution 4.0 International License.

**FIGURE 8:**
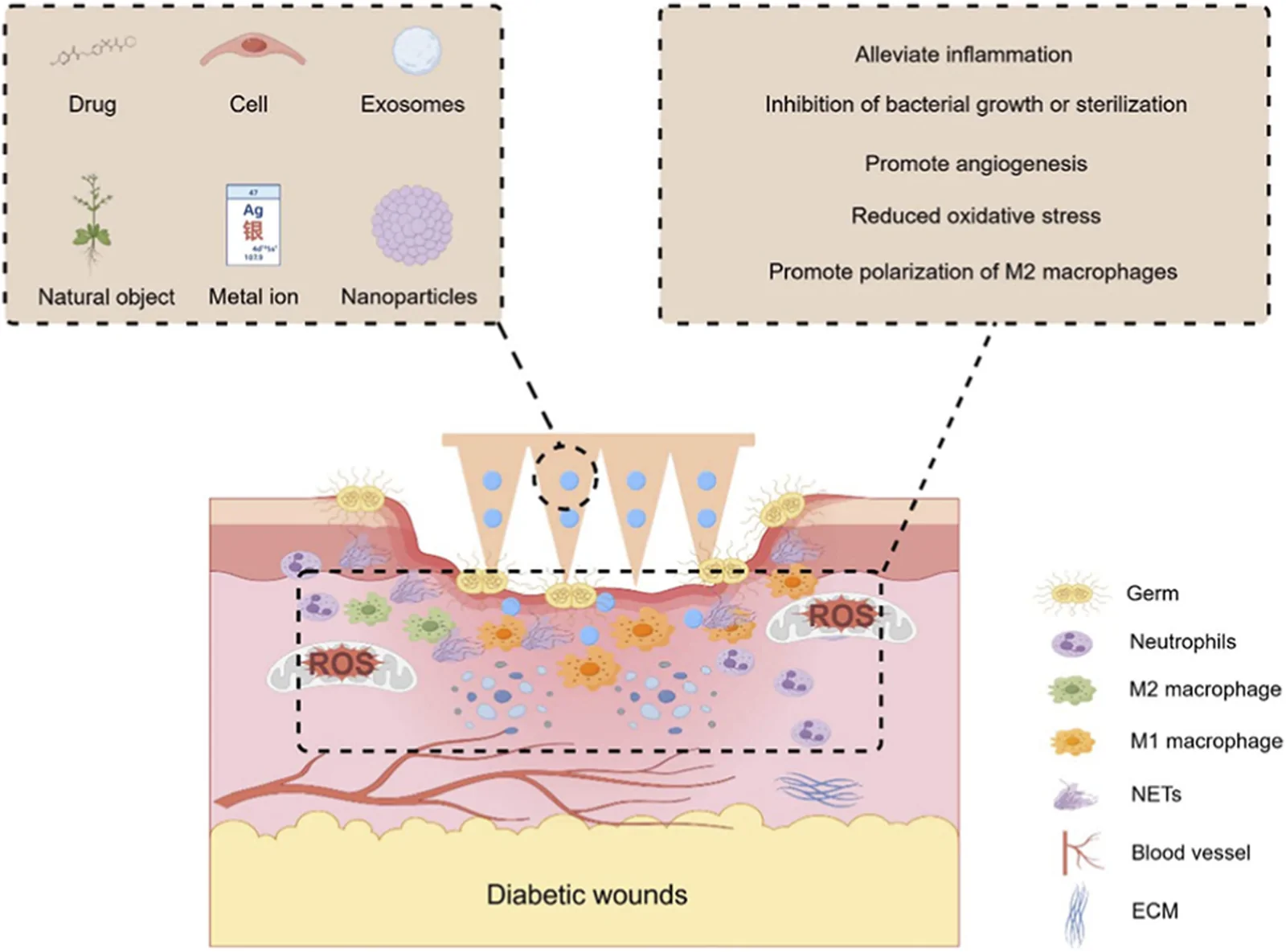
Multifunctional microneedle strategies for diabetic wound repair. The schematic illustrates potential cargoes and functions and does not imply validation in an infected diabetic wound model. Reproduced from Jian et al. (2025), subject to the applicable reuse license.

**FIGURE 9:**
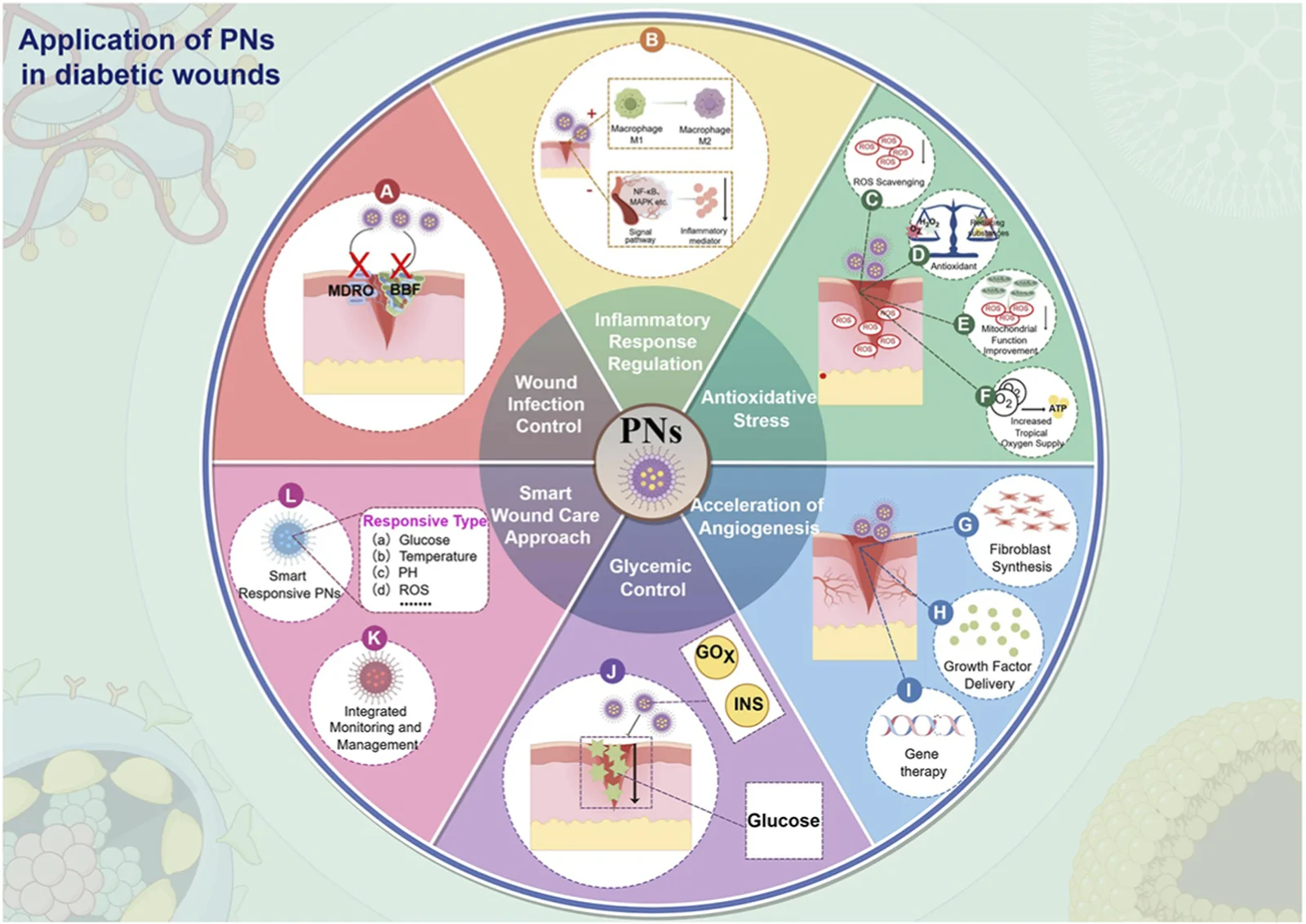
Polymeric nanomedicine strategies for diabetic wound healing. The general nanomedicine mechanisms shown are indirect design evidence and not specific proof of HA efficacy in infected DFUs. Reproduced from Chen et al. (2025b) under the CC BY-NC 4.0 license.

**FIGURE 10:**
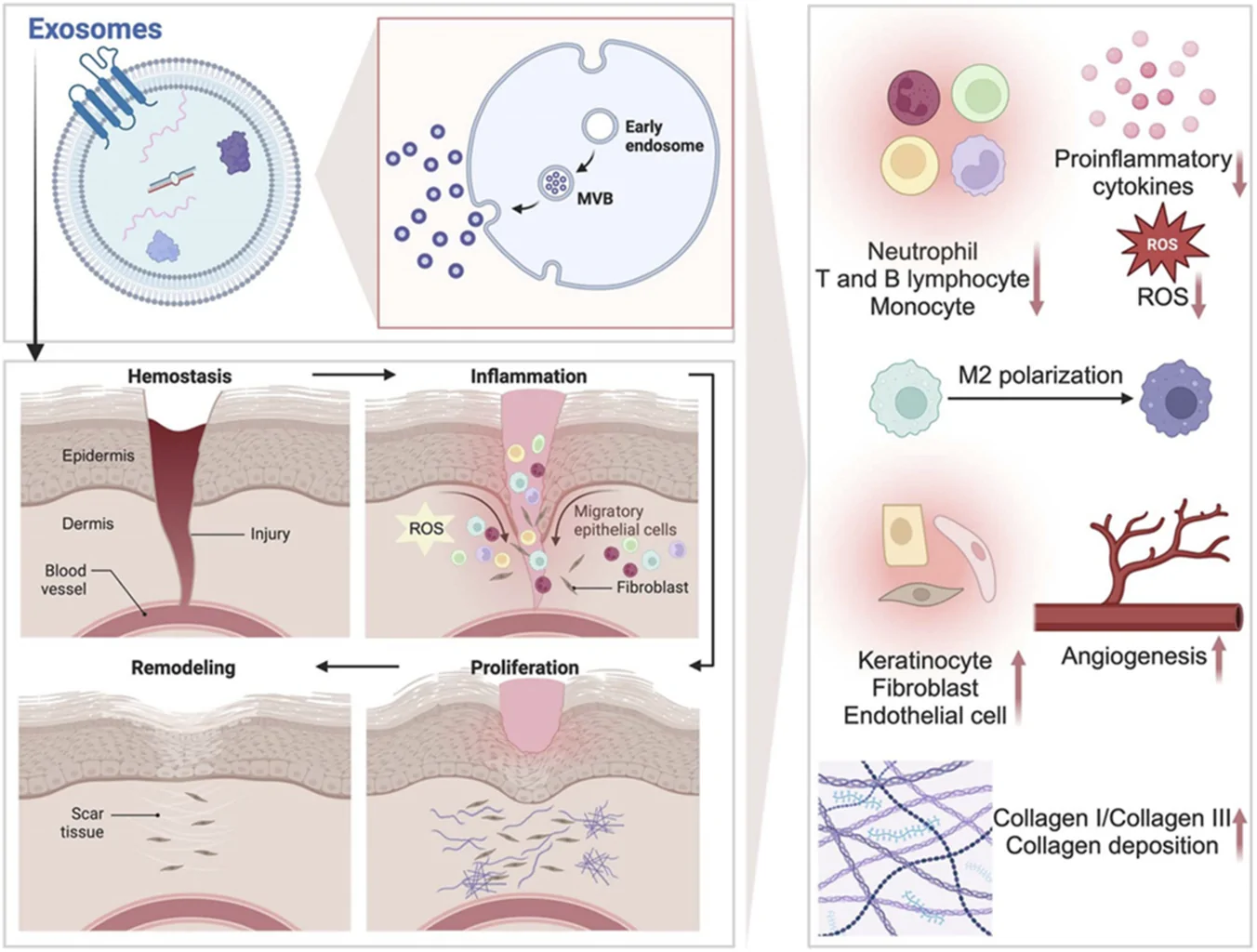
Reported mechanisms of extracellular-vesicle-mediated diabetic wound repair. These mechanisms arise from the vesicle cargo; an HA matrix may improve retention and presentation. Reproduced from Jin et al. (2025) under the Creative Commons Attribution 4.0 International License.

**FIGURE 11:**
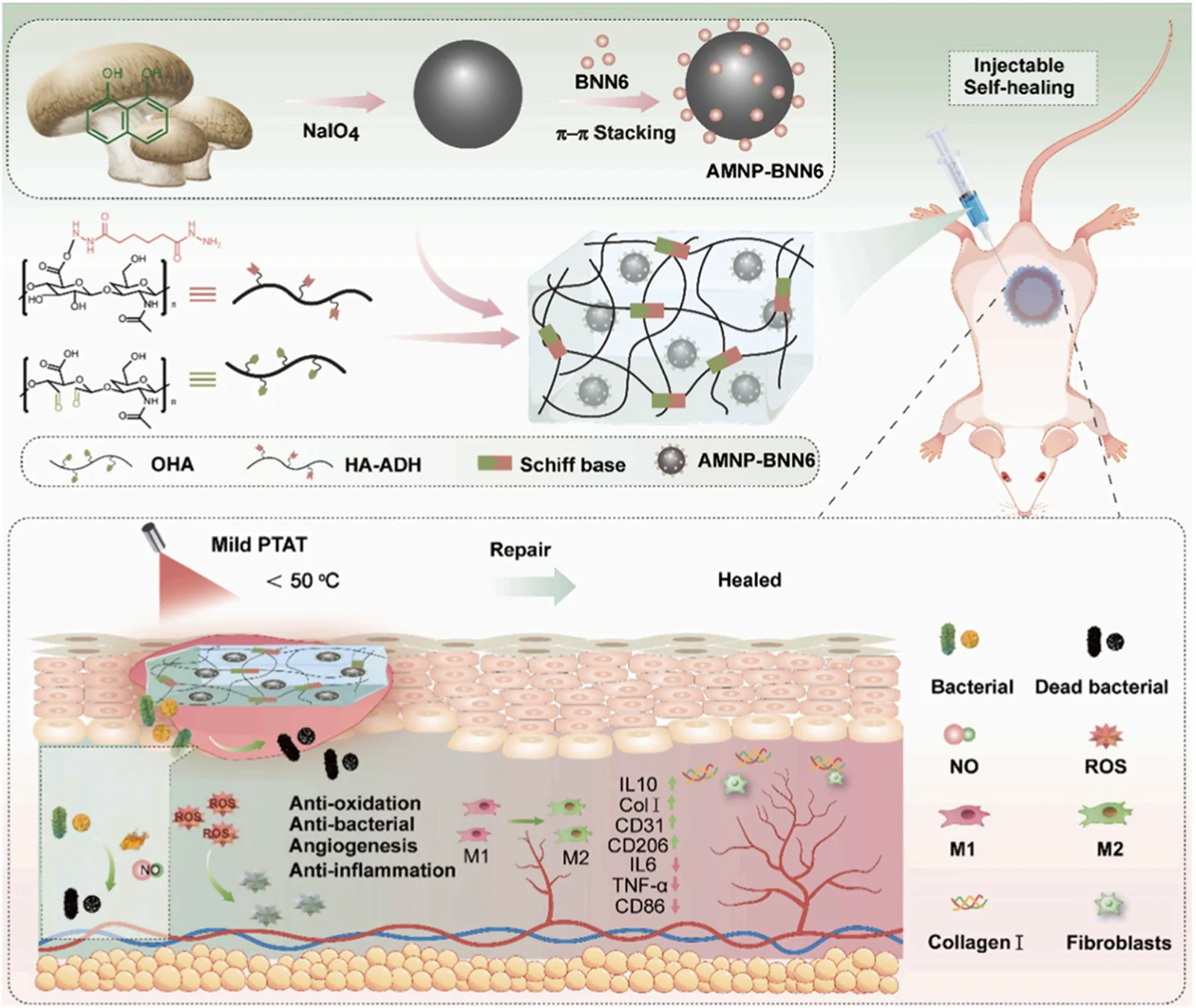
Preparation and application of the multifunctional Gel-HAB composite. The AMNP-BNN6 module exerts photothermal, ROS-scavenging and nitric oxide release functions, whereas the HA-containing dynamic network supplies the local matrix and retention. The figure depicts preclinical evidence from an explicit infected diabetic wound model. Reproduced from He et al. (2024) with permission from Elsevier.

### 3.1 Hyperglycemic injury and inflammatory amplification

AGE-RAGE signaling also intersects with redox imbalance and proteolysis: NF-κB-associated cytokines can increase MMP expression, while oxidative injury reduces cell migration and matrix synthesis. A material that claims to modify this axis should therefore report pathway-specific measures such as RAGE expression, NF-κB nuclear translocation and the levels of inflammatory mediators together with fibroblast/keratinocyte migration, matrix organization and epithelial continuity. Wound closure alone cannot determine whether the metabolic–inflammatory mechanism was altered.

Persistent hyperglycemia promotes the accumulation of advanced glycation end products and RAGE/NF-κB signaling, increasing the expression of proinflammatory mediators and impairing fibroblast, keratinocyte and endothelial cell function (Burgess et al., 2021; Lin et al., 2025d; Wang et al., 2017). In infected tissues, this metabolic–inflammatory state can compromise host defense and reinforce oxidative stress, poor granulation and delayed epithelial repair.

HA hydrogels can retain anti-inflammatory agents, antioxidants or extracellular vesicles locally, but the notable biological activity is typically mediated by the cargo. In a noninfected diabetic wound model, an oxidized HA-containing hydrogel carrying RAGE- and MMP-9-directed components reduced NF-κB-, ROS- and MMP-9-associated readouts (Banerjee et al., 2024b). This finding supports the mechanistic plausibility of local delivery but does not establish control of infected DFUs.

### 3.2 Infection and mature biofilms

Coordination with clinical antimicrobial stewardship is also necessary for infection-specific design. A local platform may increase tissue exposure or sustain drug release, but antibiotic choice, pathogen susceptibility, systemic therapy, source control and perfusion remain crucial decisions. Photothermal-, photodynamic- and metal-based strategies should be considered on the basis of therapeutic selectivity because a reduction in colony count accompanied by endothelial or fibroblast injury may delay repair. Clinically meaningful evidence therefore couples microbial, inflammatory, safety and tissue quality outcomes.

Infected DFUs may contain polymicrobial communities embedded in an extracellular polymeric matrix. Biofilm physiology reduces antimicrobial penetration and immune clearance and can sustain inflammatory signaling (Afonso et al., 2021; Macdonald et al., 2021; Theodorakopoulos and Armstrong, 2025). Material testing should distinguish the prevention of initial biofilm attachment from the disruption of established biofilms and the suppression of their regrowth.

HA can improve the local retention and contact of antimicrobial cargo, whereas the bactericidal activity generally originates from encapsulated antibiotics, cationic polymers, metal ions, photothermal modules or nitric oxide. Direct evidence requires an explicit infected diabetic wound model with viable microbial and tissue repair endpoints. *In vitro* mechanistic evidence or sterile diabetic wound closure data should not be labeled infection control.

### 3.3 Excessive ROS levels and redox imbalance

The distinction between a ROS-responsive matrix and an antioxidant material is important. Oxidation-sensitive bonds may merely trigger cargo release, whereas a polyphenol, nanozyme or other additive performs chemical scavenging. Controls should compare the unloaded network, free cargo and complete formulation and measure drug release at wound-relevant ROS levels. Otherwise, a response detected at an artificially high *in vitro* oxidant concentration may not predict behavior in spatially heterogeneous tissue.

ROS participate in antimicrobial defense and repair signaling when their levels are controlled, but persistent excess ROS damage lipids, proteins and DNA, impair reparative cells and increase inflammation and MMP activity (Cai et al., 2023; Deng et al., 2021). The appropriate design objective is the restoration of redox homeostasis, not the indiscriminate elimination of ROS.

ROS-responsive HA networks can incorporate oxidation-sensitive bonds to alter matrix degradation or cargo release, while polyphenols, catechol groups, cerium oxide, manganese dioxide and other related cargo provide much of the observed antioxidant activity (Shi et al., 2024; Xu et al., 2022). Evaluations should therefore combine ROS, SOD, glutathione and lipid peroxidation measures with those of microbial defense, cell viability, angiogenesis and matrix quality.

### 3.4 Immune dysregulation

Treatment timing is especially important after infection. Early neutrophil and macrophage activity contributes to pathogen clearance; suppressing these responses before the source is controlled may be harmful. The objective is not a permanent shift to an M2-like state but the restoration of a coordinated sequence from defense to resolution and repair. Longitudinal sampling and functional phagocytosis or microbial clearance assays are more informative than a single terminal marker panel is.

Macrophages contribute to early pathogen clearance and later to inflammation resolution, vascular support and matrix remodeling. Diabetes and infection can prolong inflammatory states and impair the coordinated transition to repair (Wu et al., 2022; Al Sadoun, 2022; Clayton et al., 2024). The M1/M2 dichotomy is an incomplete simplification; CD86, iNOS, CD206 or Arg-1 measurements should be complemented by phagocytosis, cytokine spectra, temporal profiling and tissue-level function. Single-cell studies revealed multiple macrophage, immune, fibroblast and epidermal states in diabetic wounds; these datasets strengthen the pathobiological framework but do not by themselves validate any HA treatments (Ma et al., 2023; Gan et al., 2025; Liu et al., 2025).

HA-CD44 interactions may influence immune cell adhesion and signaling, while HA matrices can retain paeoniflorin, cytokines or extracellular vesicles. In a noninfected diabetic wound model, a paeoniflorin-loaded HA hydrogel altered the expression of macrophage-associated markers and improved repair (Yang et al., 2021). These data provide *in vitro* and preclinical mechanistic evidence for immunomodulation but are not proof that premature immune suppression is safe during active infection.

### 3.5 Ischemia, hypoxia and impaired vascular repair

Hypoxia can be both a physiological signal and a pathological constraint. Transient HIF signaling supports wound repair, whereas persistent severe ischemia limits cell survival and drug delivery. Therefore, analyses of the dose, duration and diffusion are necessary when evaluating oxygen-generating and nitric oxide-releasing materials. An increase in vessel staining intensity within a poorly perfused wound is not a substitute for evidence that the new vessels are mature, connected to the circulation and capable of improving tissue oxygenation.

PAD, microvascular dysfunction and unstable HIF-1alpha/VEGF signaling restrict oxygen, nutrient and antimicrobial delivery and impair fibroblast, endothelial and epithelial repair (Catrina and Zheng, 2016; Zhu et al., 2024; Fitridge et al., 2024). Angiogenesis-oriented biomaterials are biologically plausible only after the severity and reversibility of macrovascular perfusion deficits have been assessed.

HA hydrogels can prolong the local presentation of VEGF, bFGF, PDGF, nitric oxide donors, copper ions or extracellular vesicles, but these cargoes provide the direct angiogenic or vasoregulatory effects. CD31 or VEGF expression alone does not demonstrate restored perfusion. Vessel maturity, alpha-SMA coverage, tissue oxygenation, functional perfusion and persistence measurements are needed (Rai et al., 2023).

### 3.6 Proteolysis, ECM degradation and delayed re-epithelialization

Matrix repair is a downstream integrator of infection control, redox balance, immune resolution and perfusion. MMP-responsive materials may exploit pathological protease activity, but overly rapid carrier cleavage can eliminate the scaffold before repair can be achieved, whereas excessive crosslinking can impede remodeling and epithelial migration. Degradation products, pore evolution and cell infiltration should therefore be characterized alongside conventional collagen staining.

Excessive MMP-2 and MMP-9 activity leads to the degradation of collagen, fibronectin, laminin and growth factors, weakening cell‒matrix interactions and decreasing epithelial continuity (Fu et al., 2022; Chen et al., 2023; Zhang et al., 2023). Infection-associated proteases, ROS and inflammatory cytokines can further accelerate this process.

High glucose can also impair re-epithelialization through epigenetic enhancement of FOXO1–MMP9 signaling; inhibition of the related epigenetic changes rescued keratinocyte migration and wound closure in diabetic skin and mucosal wound models. This mechanistic context is independent of HA, not direct evidence of the effects of an HA material (Yang et al., 2024).

HA hydrogels and nanofibers can reproduce certain hydration or fibrous features of the ECM, and MMP-responsive linkages can couple proteolysis to degradation or cargo release. Evidence should include MMP activity, TIMPs expression, collagen composition and alignment, fibronectin preservation, epithelial continuity and sustained wound closure rather than the wound area alone (Lin, Hu, et al., 2025; Hosty et al., 2024). Table 1 links these pathological axes to design objectives and evidence boundaries.

**TABLE 1:** Pathological microenvironment of infected diabetic foot ulcers and the corresponding design logic for HA-based biomaterials.

Pathological target	Major abnormalities and repair consequences	HA-based design objective	Representative platform or functional module	Recommended mechanistic and repair indices	Evidence boundary/clinical caveat	Representative reference(s)
Hyperglycemic injury and AGE–RAGE/NF-κB amplification	AGE accumulation and sustained RAGE/NF-κB activation increase inflammatory cytokine levels and impair fibroblast, keratinocyte and endothelial cell function	Attenuate local metabolic–inflammatory amplification and protect reparative cells	HA hydrogels delivering anti-inflammatory agents, antioxidants, extracellular vesicles or immunomodulators	AGE/RAGE; NF-κB p65; TNF-α, IL-1β, IL-6; cell migration; granulation; re-epithelialization	Local materials do not correct systemic hyperglycemia; evidence is mainly from sterile rodent wounds	(Lin et al., 2025d)
Polymicrobial infection and mature biofilms	Mature biofilms reduce antimicrobial penetration and immune clearance, sustaining inflammation, regrowth and recurrence	Disrupt established biofilms and suppress regrowth while preserving reparative cell function	HA matrices combined with antibiotics, peptides, chitosan, metal ions or photothermal, photodynamic or NO modules	Tissue CFUs; mature biofilm architecture; regrowth; infection resolution; repeat debridement; cytocompatibility	Direct claims require an explicitly infected diabetic model; remains adjunctive to source control	(Theodorakopoulos and Armstrong, 2025)
Excessive ROS and redox imbalance	Persistent ROS damage reparative cells, increase MMP activity and impair angiogenesis and collagen organization	Restore redox homeostasis without eliminating physiological ROS-dependent defenses and signaling	ROS-responsive HA networks containing polyphenols, catechol, dopamine, selenium, CeO_2_ or MnO_2_ modules	ROS; SOD; GSH; MDA; Nrf2/HO-1; MMP-2/MMP-9; cell viability; angiogenesis	Antioxidant effects are often cargo mediated and must be interpreted against host defense and temporal ROS biology	(Deng et al., 2021)
Persistent inflammatory macrophage responses	Failure to resolve inflammation results in poor granulation and restricted ECM remodeling	Promote resolution-oriented immune regulation and functional repair-associated macrophage responses	HA matrices containing paeoniflorin, cytokines, anti-inflammatory agents or macrophage-derived extracellular vesicles	CD86/iNOS; CD206/Arg-1; phagocytosis; cytokine profile; temporal changes; granulation quality	The M1/M2 dichotomy is incomplete; marker shifts require functional and temporal validation	(Wu et al., 2022) (Clayton et al., 2024)
Ischemia, hypoxia and impaired angiogenesis	Low perfusion and impaired HIF-1α/VEGF signaling restrict oxygen delivery, vessel formation and epithelial repair	Support angiogenesis, vascular maturation and oxygen availability after formal vascular assessment	HA matrices delivering VEGF, bFGF, PDGF, NO donors, copper ions, oxygen-releasing components or vesicles	CD31; α-SMA; VEGF; vessel architecture; tissue perfusion; oxygenation; granulation thickness	Local materials cannot compensate for severe untreated PAD or chronic limb threatening ischemia	(Fitridge et al., 2024)
Aberrant MMP activity, ECM degradation and delayed re-epithelialization	Excessive activation of MMP-2/MMP-9 leads to the degradation of collagen, fibronectin and growth factors and reduces epithelial continuity	Preserve matrix structure, maintain biological cues and support cell migration	ECM-mimetic HA hydrogels or nanofibers; collagen/gelatin/chitosan composites; MMP-responsive networks	MMP/TIMP; collagen I/III; collagen alignment; fibronectin preservation; epithelial continuity; sustained wound closure	A short-term reduction in wound area does not establish high-quality or sustained healing	(Fu et al., 2022) (Chen et al., 2023)

The listed strategies define directions for biomaterial development and evidence evaluation. They are research hypotheses rather than substitutes for debridement, antimicrobial treatment, offloading, vascular assessment or revascularization.

Abbreviations: AGE, advanced glycation end product; bFGF, basic fibroblast growth factor; DFU, diabetic foot ulcer; ECM, extracellular matrix; GSH, glutathione; HA, hyaluronic acid; HIF-1α, hypoxia-inducible factor-1α; MDA, malondialdehyde; MMP, matrix metalloproteinase; NF-κB, nuclear factor-κB; NO, nitric oxide; PAD, peripheral arterial disease; PDGF, platelet-derived growth factor; RAGE, receptor for advanced glycation end products; ROS, reactive oxygen species; SOD, superoxide dismutase; TIMP, tissue inhibitor of metalloproteinase; VEGF, vascular endothelial growth factor.

## 4 HA biological and engineering basis

### 4.1 Comparative positioning of HA

Comparative positioning suggests specific rather than universal indications for HA. HA is most compelling when hydrated ECM mimicry, receptor interactions and adaptable local delivery are central. Chitosan may be preferred when intrinsic cationic antimicrobial effects are the main requirement; alginate when absorptive exudate control is predominant; collagen or gelatin when abundant adhesion motifs are needed; and synthetic networks when tightly reproducible mechanical properties and degradation behavior are most important. Hybridization is often rational, but it makes component attribution and manufacturing control more demanding.

HA is a linear anionic glycosaminoglycan composed of D-glucuronic acid and N-acetyl-D-glucosamine. Its hydrophilicity supports hydrated, viscoelastic matrices; its ECM association and receptor binding distinguish it from purely inert carriers; and its modifiable backbone permits the formation of dynamic, adhesive, photocrosslinked and stimulus-responsive networks (Chang et al., 2024; Wu et al., 2024; Valachová et al., 2024). These are platform advantages, not evidence of universal superiority. HA often requires chitosan, collagen/gelatin, silk, PCL, PEG or other components to compensate for its weak mechanical strength, rapid degradation, difficulty with electrospinning or limited intrinsic antimicrobial activity.

### 4.2 ECM mimicry and chemical modifications

Modification can also affect HA recognition, degradation and safety. The degree of substitution determines the residual availability of native functional groups, the crosslinking density alters diffusion and fragmentation, and sterilization may change the molecular weight or mechanical behavior. Reporting data for the nominal starting polymer and crosslinker only is insufficient; poststerilization chemistry, rheology, swelling, release and degradation of the final product should be characterized.

Carboxyl and hydroxyl groups provide the main sites for oxidation, methacrylation, amidation, esterification, catechol grafting and sulfation. These modifications affect gelation, wet tissue adhesion, mechanical activity, swelling, degradation and cargo release (Bokatyi et al., 2024; Hintze et al., 2022; Burdick and Prestwich, 2011). Chemical responsiveness is only translationally useful when the trigger thresholds and kinetics match spatially and temporally heterogeneous wounds.

### 4.3 Receptor signaling, molecular weight and contamination-sensitive interpretation

CD44 is involved in immune cell trafficking, keratinocyte migration, endothelial responses and fibroblast behavior; RHAMM/HMMR contributes to motility, cytoskeletal organization and repair signaling (Guan et al., 2025; Tolg et al., 2014). These interactions support the function of HA as a cell-interactive matrix. They do not imply that every HA-containing composite has intrinsic antimicrobial, antioxidant or angiogenic activity.

High-molecular-weight HA is commonly associated with homeostatic and anti-inflammatory effects, whereas some low-molecular-weight fragments have been linked to TLR2/TLR4-associated inflammation. This pattern is context dependent and influenced by fragment size, purity, concentration, cell type, receptor expression, exposure duration and degradation kinetics (Tavianatou et al., 2019; Garantziotis and Savani, 2019). Endotoxin-free HA fragments have failed to reproduce some previously reported inflammatory responses, demonstrating that endotoxin or protein contaminants can be mistaken for intrinsic HA activity (Dong et al., 2016). Mechanistic claims must therefore be supported by defined fragment distributions, endotoxin testing results, purification controls and appropriate receptor or pathway experiments.

## 5 HA material platforms: pathology-directed design, component attribution and evidence

HA-based platforms are evaluated here according to three questions: which functions are supplied by the HA matrix, which are supplied by the incorporated cargo or copolymers, and do component-level controls demonstrate an independent contribution from HA. Evidence is further classified as arising from explicit infected diabetic wound models, noninfected diabetic wound models, *in vitro* mechanistic studies, or clinical DFU populations in which infection status was not reported or stratified.

### 5.1 HA hydrogels

Adhesive and self-healing labels should be linked to application-relevant tests. Lap shear on healthy tissue under dry conditions does not reproduce a plantar ulcer exposed to exudate, repeated deformation or dressing changes. Wet adhesion, cyclic shear, fatigue recovery, fluid uptake, drug loss and removal without tissue injury should be assessed. For highly exudative or repeatedly debrided wounds, easy drainage and access may be more important than maximal residence time.

HA hydrogels offer a high water content, wound conformity and tunable crosslinking. The HA network can support matrix formation, injectability, self-healing behavior, adhesion, degradation control and local retention, while the incorporated cargo usually exert the specific antimicrobial, antioxidant or growth-promoting effects. Mechanical strength, swelling, exudate transport, drainage and repeat access must be balanced. Injectable formats have been proposed for application to certain irregular postdebridement defects, but evidence that a hydrogel can replace the outcomes of drainage or debridement of deep infections is lacking.

Stimulus-responsive hydrogels exploit acidic pH or high levels of glucose, ROS, MMPs or enzymes to alter the network structure or induce cargo release. In a noninfected diabetic wound model, the OHA-PBA/CMCS/PA platform exploited acidic and hyperglycemic conditions to trigger the cleavage of dynamic imine and boronate interactions to release proanthocyanidins; the degradation of the polymer network was controlled, while the polyphenol supplied much of the detected antioxidant activity (Zhang et al., 2025).

In noninfected diabetic wound models, glucose- or ROS-responsive HA hydrogels containing catechin, tannic acid, curcumin or related components have reduced oxidative and inflammatory readouts and improved wound closure (Xu et al., 2022; Wang et al., 2024; Shi et al., 2024). These studies support responsive delivery and cytoprotection, but antibacterial assays performed in a noninfected diabetic model do not establish infection treatment. Redox studies should also show that physiological host defense is preserved.

Other HA composites provide broad indirect evidence for inflammation, angiogenesis and metabolic regulation. Dynamic HA networks coordinating H_2_S and Zn^2+^, dopamine-functionalized HA–recombinant type III collagen hydrogels, Zn^2+^/dexamethasone-loaded HAMA systems, and HA-PEI/siRNA-29a hydrogels were evaluated in diabetic or full-thickness wound models without clear establishment of a wound *in vivo*. These studies support platform design and regenerative readouts, but any antimicrobial, anti-inflammatory or angiogenic effects should be attributed to the complete formulation unless HA-only and cargo-only controls are included to isolate individual contributions (Chen et al., 2024; Wang et al., 2024; Yang et al., 2025; Yang et al., 2021).

More direct infection evidence is available for certain systems. In an explicit infected diabetic wound model, a modified HA hydrogel combined phenylboronic acid-mediated bacterial capture with quaternized chitosan-mediated bacterial killing (Lan et al., 2024). HA/HAMA formed the local matrix and capture interface, whereas the cationic chitosan derivative killed the pathogen upon contact. Other HA systems containing photothermal modules, silver, bacteriocins or metal ions have been reported to have antimicrobial effects, but mature biofilms, regrowth, resistance and host cell selectivity remain incompletely assessed (Yang et al., 2023; Zhai et al., 2024; Yin et al., 2024). Recent studies of infected diabetic wounds have reported self-evolving, dual-drug and pH-responsive metformin-releasing HA/hyaluronate hydrogels. Because each system contains multiple components, their efficacy is a formulation-level result unless HA-only and cargo-only controls are included to isolate individual contributions (Liu et al., 2026; Qiu et al., 2025; Zheng et al., 2026).

Reports of two additional HA-based composites provide direct evidence after infection. A lauric acid-mediated gelatin/oxidized HA hydrogel was evaluated in a diabetic wound model of MRSA infection, and an injectable HA-ADH/oxidized dextran hydrogel carrying LAP@W379 nanoparticles achieved pH-responsive antimicrobial peptide delivery and biofilm elimination in MRSA-infected diabetic wounds. Notably, the antibacterial and immunoregulatory effects of both systems should be attributed to the complete formulation, not HA alone (Yang et al., 2025b; Zhao et al., 2024).

### 5.2 HA nanofibers

Fiber diameter, porosity and alignment affect cell migration, fluid transport and mechanical behavior, whereas the HA content and crosslinking affect hydration and persistence. Clinical translation requires reproducible electrospinning results, control of residual solvent, sterilization, packaging and product stability. Antimicrobial additives should not obscure the primary evidence: does the fibrous architecture improve epithelial and matrix repair under clinically relevant wet conditions?

Electrospun nanofibers reproduce certain features of the fibrous ECM and offer a high surface area for local delivery. Electrospinning pure HA is difficult, and HA is unstable in water; thus, PCL, PLGA, collagen, gelatin, chitosan or silk are frequently incorporated to increase spinnability and tensile strength (Humaira et al., 2022; Valachová et al., 2022). HA contributes to hydration and matrix interactions, but the biological and mechanical contributions of each component of the blend should be separated.

Sulfated HA/collagen nanofibers have shown preliminary repair activity in a noninfected diabetic wound model (Zhou et al., 2024). Their potential for use in shallow wounds with manageable exudate has been inferred from their material properties, although their performance in infected plantar tissues, wet mechanical stability, sterilization and scale-up remain unproven.

### 5.3 HA microneedles

Microneedles require an anatomically realistic target. Insertion into intact porcine skin demonstrates mechanical properties but not performance at a macerated, callused, inflamed or partly necrotic wound edge. Drug distribution may be nonuniform, and needle fracture or incomplete dissolution may introduce retained material. Studies should distinguish delivery to intact periwound skin from insertion into the ulcer bed and justify the chosen tissue compartment.

Dissolving or hydrogel-forming HA microneedles can deliver cargo to superficial viable tissue or the wound edge. The proposal that they bypass protein-rich exudate, hyperkeratosis or early biofilm layers is a testable engineering concept, not a clinically established indication. Deep infection, extensive necrosis, osteomyelitis, heavy exudate and large postdebridement dead spaces are inappropriate scenarios for microneedle use without source control and the presence of viable tissue.

In a diabetic wound model in which infection was not clearly established, HA/PVA microneedles carrying resveratrol micelles and gentamicin produced antibacterial, antioxidant and inflammatory effects (Li et al., 2024). Baek et al. (2024) provided preliminary *in vitro* and *ex vivo* mechanistic evidence for microneedle fabrication, penetration through porcine skin or polymer membranes and epidermal growth factor delivery; this system was not tested in an infected diabetic wound animal model. These stages should not be presented as equivalent. More recent core–shell, engineered yeast and gallium–tannic acid HA microneedles were evaluated in infected diabetic wound models; their antimicrobial and immunomodulatory effects resulted from the complete loaded system and should not be attributed to the HA needle matrix alone (Wei et al., 2025; He et al., 2025; Wang et al., 2026).

Two additional HA microneedle studies illustrate why the evidence stage and component attribution must remain explicit. HAMNs@AgNPs-Ins combined an HA microneedle with silver nanoparticles and insulin, whereas ZCO-HA combined the HA needle matrix with a cerium/zinc nanomaterial. Both reported *in vitro* antibacterial activity and the ability to heal diabetic wounds, but an infected *in vivo* wound model was not explicitly established; these systems are therefore treated here as unconfirmed evidence of infection, and their antibacterial effects should not be attributed to HA alone (Chen et al., 2025; Yang et al., 2023).

Future studies should report the insertion force and success, dissolution, delivered dose, tissue recovery, pain, wound edge integrity and performance in the presence of exudate and under plantar pressure and cyclic shear. The effects of the antimicrobial cargo must be separated from the structural role of the HA needle matrix.

### 5.4 HA nanoscale delivery systems

Free nanoparticles may be cleared with exudate or exhibit unpredictable penetration; thus, embedding them in a hydrogel, fiber or microneedle can improve retention but adds another transport barrier. Particle size, charge, HA density, the presence of a protein corona, matrix mesh size and degradation jointly determine release and uptake. CD44 targeting should therefore be supported by receptor blocking or competing ligand controls and by distribution studies in heterogeneous wound tissues. Studies investigating HA of various molecular weights have shown that uptake and targeting depend on more than receptor expression. Recent evidence is derived mainly from nanoparticles or extracellular vesicles outside diabetic wounds and should therefore be treated as engineering evidence rather than proof of DFU targeting (Chiesa et al., 2022; Della Sala et al., 2022; Kyykallio et al., 2026). Polymeric nanomedicine strategies have also been reviewed in the context of diabetic wound healing (Chen et al., 2025b).

HA-containing or HA-coated nanoparticles, nanogels, liposomes and micelles can protect unstable cargo, regulate its release and support context-dependent CD44-mediated uptake (Salathia et al., 2023; Kwon et al., 2019). HA acts primarily as a hydrophilic carrier or surface ligand; the encapsulated drug exerts the principal pharmacological effect. CD44 expression in isolated cells does not establish selective targeting in heterogeneous, protein-rich, exudative wounds.

Curcumin-loaded HA-conjugated PLGA nanoparticles provide *in vitro* and inflammatory disease proof-of-concept evidence for HA-mediated delivery rather than direct evidence of diabetic wound wounds (Hlaing et al., 2022). Clinically relevant studies need to investigate tissue distribution, cellular uptake, protein corona effects, retention, clearance, systemic exposure and safety in claim-matched infection models.

### 5.5 HA matrices containing extracellular vesicles, growth factors or cells

The biological cargo also changes the regulatory identity of the product. The final formulation may combine the attributes of the device, drug and biological product, with potency depending on the donor, culture, isolation and loading. Release assays should quantify intact active cargo rather than total protein or particle number alone. Comparators should include free cargo and unloaded HA samples so that improved retention can be distinguished from the biological potency of the payload.

HA hydrogels can reduce the dispersion and proteolysis of extracellular vesicles or growth factors and provide sustained local release. The vesicle or protein cargo, not HA alone, exerts most of the immunomodulatory, angiogenic or epithelial signaling. In noninfected diabetic wound models, HA-containing matrices loaded with mesenchymal stromal cell or macrophage-derived vesicles have exhibited improved endothelial, fibroblast and repair readouts (Banerjee et al., 2024a; Yu et al., 2025).

Growth factor-loaded matrices face a narrow design window: insufficient exposure is ineffective, whereas excessive or poorly timed delivery may result in leaky immature vessels, increased exudate or off-target effects (Uccelli et al., 2019; Zheng et al., 2023). Vesicle products add variations in source, isolation, potency, batch, storage and regulatory variability (Welsh et al., 2024). Their proposed clinical niche is poor granulation or delayed epithelialization after infection control and adequate perfusion have been achieved, not during uncontrolled infection.

### 5.6 Cross-platform synthesis and the Gel-HAB attribution as an example

A related design question is whether multifunctionality should be parallel or sequential. Simultaneous antimicrobial, anti-inflammatory and angiogenic activity is attractive conceptually, but the optimal dose and timing of each function may differ. A stage-responsive system that prioritizes source control early and regenerative signals later may reduce antagonistic interactions and improve translatability. This hypothesis requires time-resolved release, microbial, immune and repair data rather than a single terminal wound closure comparison.

The Gel-HAB system illustrates why component attribution is necessary. In an explicit infected diabetic wound model, the AMNP-BNN6 module provided mild photothermal-mediated antibacterial activity, ROS scavenging and near-infrared-triggered nitric oxide release; the dynamic HA network more directly promoted matrix formation, injectability/self-healing behavior and local retention (He et al., 2024). The response of the entire composite cannot be attributed to HA without controls that compare the HA matrix, active module and complete system.

Table 2 therefore distinguishes reported evidence from scenarios inferred from engineered properties. More functions are not automatically better: greater antimicrobial or photothermal efficacy may injure host cells; excessive ROS removal may impair defenses; premature immune suppression may compromise pathogen clearance; and dense crosslinking may improve retention while restricting drainage, degradation and cell migration.

**TABLE 2:** Major HA-based tissue-engineered platforms, component attribution and evidence-bound applicability to infected diabetic foot ulcers.

Material platform	HA contribution	Cargo/copolymer contribution	Principal advantage(s)	Potential applications and evidence status	Key limitations	Representative reference(s)
Injectable/adhesive HA hydrogels	Dynamic matrix formation, wound conformity, wet tissue adhesion, degradation control and local retention	Antimicrobial, antioxidant, immunomodulatory or regenerative activity depending on the incorporated therapeutic component	Conforms to selected irregular defects and prolongs local drug delivery	Proposed application: certain irregular postdebridement defects after adequate source control; direct clinical validation for deep infected cavities is lacking	Swelling, weak mechanical properties, drainage requirements and the need for repeat wound access	(Duan et al., 2024)
Stimulus-responsive/dynamically crosslinked HA hydrogels	Trigger-sensitive network degradation, structural rearrangement or release in response to pH, glucose, ROS, MMPs or enzymes	Polyphenols, drugs, proteins or particles provide most of the specific antioxidant, anti-inflammatory or regenerative effects	Couples disease-associated cues to controlled release or network remodeling	Indirect evidence is derived mainly from noninfected diabetic wound models; phenotype-specific use remains a design hypothesis	Trigger thresholds and release kinetics may not match spatially and temporally heterogeneous human wounds	(Chhillar and Jaiswal, 2025) (Zhang, Zhang, et al., 2025)
Antimicrobial/antibiofilm HA hydrogels	Local matrix formation, wound contact, bacterial capture where specifically engineered, and cargo retention	Cationic polymers, antibiotics, metal ions, photothermal/photodynamic modules or NO donors supply most of the direct antimicrobial effects	Provides high local exposure to antimicrobials while allowing combination with repair-supportive functions	Direct preclinical infection model evidence exists for certain systems after debridement/source control	Mature biofilm, regrowth, resistance evolution, host cell toxicity and external energy requirements remain incompletely assessed	(Lan et al., 2024)
Nanofibers	Hydration, ECM-associated biological interface and selected matrix-mimetic properties	PCL/PLGA/collagen/chitosan/silk commonly increases spinnability and mechanical strength; the incorporated cargo provides the specific therapeutic activity	Provides fibrous support with high surface area for superficial repair and local delivery	Indirect evidence is derived mainly from noninfected diabetic wounds; infected plantar application remains unproven	Wet stability, conformity, exudate management, sterilization and scale-up	(Zhou et al., 2024)
Microneedles	Dissolving or hydrogel-forming local-delivery matrix for superficial viable tissue or wound edge delivery	Drug, growth factor or nanoparticle/micelle cargo provides the principal therapeutic effect	Enables local, staged or minimally invasive delivery	Mixed evidence stage: some diabetic wound studies exist; Baek et al. provided only *in vitro*/*ex vivo* evidence; use in infected DFUs remains hypothetical	Insertion reliability, pain, exudate control, wound edge integrity; unsuitable for deep infections or osteomyelitis without source control	(Li et al., 2024) (Baek et al., 2024)
Nanoscale delivery systems	Hydrophilic carrier or surface ligand with context-dependent CD44-associated interaction	Encapsulated drug, nucleic acid or other cargo supplies the principal pharmacological activity	Protects unstable cargo and supports combination or spatially controlled delivery	Evidence is mainly *in vitro*/indirect; proposed for scaffold-embedded local delivery rather than free suspension administration	Protein corona, tissue distribution, clearance, systemic exposure, reproducibility and manufacture	(Hlaing et al., 2022)
Vesicle/growth factor composite	Retention, protection and sustained-presentation matrix for labile biological cargo	Extracellular vesicles or growth factors supply immune, vascular, fibroblast or epithelial signaling	Protects labile cargo and prolongs local presentation	Preclinical evidence in noninfected diabetic wounds; proposed after infection control and adequate perfusion are achieved	Source, potency, purification, batch consistency, storage, immune safety, cost and regulatory complexity	(Banerjee et al., 2024a)

‘Direct’ denotes evidence from an explicit infected diabetic wound model. ‘Indirect’ denotes evidence from noninfected diabetic wound models or other mechanistic studies. The proposed applications are inferred from the engineered properties and are not established clinical indications. Photocrosslinking and self-healing are cross-cutting engineering features rather than independent therapeutic mechanisms.

Abbreviations: CD44, cluster of differentiation 44; ECM, extracellular matrix; HA, hyaluronic acid; MMP, matrix metalloproteinase; NO, nitric oxide; PCL, polycaprolactone; PLGA, poly (lactic-co-glycolic acid); ROS, reactive oxygen species.

## 6 Evidence, translational barriers and future directions

### 6.1 Evidence hierarchy and *in vitro* interpretation

Cytocompatibility should be assessed under exposure conditions that match the intended dose and release period using keratinocytes, fibroblasts, endothelial cells and relevant immune cells. Viability alone is insufficient when a material may alter migration, barrier formation, phagocytosis or vascular function. For multicomponent systems, factorial or deconvolution designs can reveal additive, synergistic or antagonistic interactions and prevent the total effect from being attributed to the HA carrier.

*In vitro* studies establish physicochemical performance, release, cytocompatibility and mechanistic plausibility. Antibiofilm claims require results from mature mono- and polymicrobial models, architecture, viable biomass and regrowth; redox claims require evidence of separation of direct scavenging from triggered release; and immune claims require functional as well as marker-based endpoints (Azeredo et al., 2017; Chen et al., 2021; Thaarup and Bjarnsholt, 2021). *In vitro* evidence cannot reproduce perfusion, neuropathy, necrosis, plantar loading or coordinated standard care. Layered collagen, epidermal reconstruction and implantable three-dimensional biofilm models provide complementary preclinical test options, but none of these models alone reproduce an infected plantar DFU (Brown et al., 2022; Cárdenas-Calderón et al., 2022; Thaarup et al., 2023).

The PEG/polyimidazolium/N-acetylcysteine hydrogel reported by Pranantyo et al. (2024) and the oxidized dextran/ε-polylysine hydrogel reported by Ma et al. (2024) did not contain HA and therefore served only as methodological comparators to evaluate infected wounds and antibiofilm effects. They should not be interpreted as direct evidence for HA-based materials. Similarly, Clayton et al. (2024) supported the biological discussion of macrophage and neutrophil dysfunction in diabetic wounds rather than the performance of a stimulus-responsive HA hydrogel.

An additional HA-independent comparator, a scallop-byssal-protein ECM-mimetic coating, was evaluated in *Staphylococcus aureus*-infected diabetic mice and can inform the infection model and outcome design; however, this is not evidence for the HA-based platform (Wang et al., 2024).

### 6.2 Animal models that match the therapeutic claim

Claim-matched endpoints are equally important. Infection studies require tissue CFU, biofilm architecture and regrowth data; redox studies require physiological oxidative measures and host defense indicators; immune studies require function and timing; vascular studies require perfusion and maturity; and matrix studies require collagen organization and epithelial quality. Reduced wound area should be treated as one outcome within this set, not as universal proof of the underlying mechanism.

No single model reproduces the full clinical pathology of DFUs. Model complexity should instead match the claim. Antimicrobial or antibiofilm claims require diabetes plus infection or mature biofilm and viable microbial endpoints. Claims of correction of ischemia require an ischemic model and functional perfusion or oxygenation. Claims of plantar adhesion, mechanical adaptation or recurrence prevention require pressure- or shear-related loading. A study focused only on matrix repair does not need an impractical all-in-one model, but its conclusions must remain within the features actually represented (Hu et al., 2022; Du et al., 2023; Couturier et al., 2023).

Table 3 separates the animal model from the infection status. Representative studies were selected to include major HA platform formats, cargo classes, direct infection model evidence, noninfected diabetic models and different stages of development. The selection is illustrative rather than systematic and is intended to expose evidence boundaries, not rank efficacy.

**TABLE 3:** Representative preclinical studies of HA-based biomaterials for diabetic wound repair according to model and infection status.

Study/reference	HA platform/cargo	Primary therapeutic claim(s)	Model	Infection status	Principal reported findings	Major translational limitations
(Yang, Song, Sun, et al., 2021)	HMW-HA hydrogel/paeoniflorin	Immune dysregulation	Diabetic mouse full-thickness wound	Noninfected	Alterations in inflammatory markers, angiogenesis, collagen deposition and epithelial repair	No biofilm, ischemia or plantar loading; cargo effect not fully separated from that of the HA matrix
(Xiong et al., 2022)	HA@MnO_2_/FGF-2/exosome hydrogel	ROS, hypoxia and regenerative signaling	Diabetic rodent wound	Not explicitly reported	Oxygen generation and timed cargo release with improved angiogenic and epithelial repair indices	Complex multicomponent system; infection relevance, component attribution and long-term safety remain unclear
(Banerjee et al., 2024b)	Oxidized-HA hydrogel/RAGE and MMP-9 inhibitors	AGE–RAGE-mediated inflammation and proteolysis	Diabetic Wistar rat wound	Noninfected	Reduced NF-κB/MMP-9-associated readouts and increased levels of repair-associated markers	No polymicrobial biofilm or plantar mechanical assessments; activity is partly cargo-mediated
(Lan et al., 2024)	HAMA-based bacterial capture matrix/quaternized chitosan	Drug-resistant infection	Infected diabetic rat wound	Infected	Bacterial capture and killing were associated with improved tissue repair	Biofilm maturity, resistance evolution and repeated debridement were incompletely reproduced
(He et al., 2024)	Dynamic HA/allomelanin–BNN6	Infection, ROS and vascular repair	Infected diabetic rodent wound model with NIR treatment	Infected	Photothermal-mediated killing, ROS scavenging and NO release improved repair-related outcomes	External irradiation, dose control and component complexity; the total effect cannot be attributed to HA.
(Shi et al., 2024)	SH-HA hydrogel/curcumin liposomes/AgNPs	ROS, bacteria and inflammation	Diabetic rodent wound	Not explicitly reported	Antioxidant, bactericidal and inflammatory readouts improved together with wound repair	No clearly defined infected diabetic model; silver exposure and degradation require further study
(Xu et al., 2022)	HAMA-PBA/catechin hydrogel	Glucose-associated ROS.	Diabetic wound	Noninfected	Glucose-triggered catechin release improved redox and repair indices	UV curing and response thresholds require clinical validation; evidence is in infected DFUs is indirect
(Wang et al., 2024)	pH/ROS/glucose-responsive HA/tannic acid	Bacteria, ROS and ECM.	Diabetic mouse wound	Not explicitly reported	Triggered release improved antibacterial and repair-related indices	Mature polymicrobial biofilms were not established; the contribution of tannic acid requires component controls
(Zhou et al., 2024)	Sulfated HA/collagen nanofiber	ECM deficiency	Diabetic wound	Noninfected	Preliminary improvement in matrix organization and tissue repair	Wet retention, exudate management and infected plantar performance remain unknown
(Li et al., 2024)	HA/PVA microneedles carrying resveratrol micelles and gentamicin	Superficial local delivery	Diabetic wound	Not explicitly reported	Combined antibacterial, antioxidant and inflammatory readouts with improved repair	Infection model unclear; insertion, exudate and deep infection relevance remain uncertain
(Baek et al., 2024)	HA microneedle/EGF.	Fabrication and delivery	*In vitro* and *ex vivo* porcine skin/membrane	Not applicable	Fabrication, penetration and release feasibility were demonstrated	No diabetic wound animal model or infection model
(Banerjee et al., 2024a)	Silk–HA hydrogel/macrophage-derived extracellular vesicles	Immune and regenerative signaling	Diabetic wound	Noninfected	Improved vesicle retention and repair-related outcomes	Vesicle source, potency, purification, batch consistency and long-term safety remain barriers

Studies were selected to represent different HA, platforms, cargoes, infection status categories and development stages. The table is narrative and illustrative rather than exhaustive or systematically graded. ‘Not reported’ indicates that an explicit infection challenge was not identified in the study description used for this review.

Abbreviations: AGE, advanced glycation end product; AgNP, silver nanoparticle; BNN6, nitric oxide donor; ECM, extracellular matrix; EGF, epidermal growth factor; FGF-2, fibroblast growth factor 2; HA, hyaluronic acid; HAMA, methacrylated HA; HMW-HA, high-molecular-weight HA; MMP, matrix metalloproteinase; NIR, near-infrared; NO, nitric oxide; PBA, phenylboronic acid; PVA, poly (vinyl alcohol); RAGE, receptor for advanced glycation end products; ROS, reactive oxygen species.

### 6.3 Clinical evidence and the infected DFU evidence gap

Clinical interventions are also heterogeneous. Some trials evaluated relatively simple HA dressings, whereas others used HYAFF scaffolds, autologous fibroblasts or platelet-rich fibrin. In the latter studies, a positive result would demonstrate the effect of the complete intervention, not HA alone. Older trials remain informative for feasibility and healing signals, but changes in infection care, offloading, vascular practice and reporting standards restrict direct comparisons.

HA dressings and derivatives have been evaluated in chronic wounds and DFUs, and some small trials report faster wound closure or greater healing proportions. Systematic reviews remain limited by differences in products, comparators, cointerventions, wound severity and risk of bias (Roehrs et al., 2023; Yao et al., 2025). Almost all published HA dressing trials include general DFU populations. Very few explicitly enroll patients with infected DFUs, stratify biofilm burden or document baseline perfusion in a manner that permits infection-specific analysis.

A broader network meta-analysis of 38 randomized trials across skin substitutes, biomaterials and topical agents supports a general DFU healing signal for several intervention classes, but it does not establish infection-specific efficacy or an independent effect of HA; it therefore informs the breadth of clinical evidence rather than validating HA as a treatment for infected DFUs (Chen et al., 2024).

Accordingly, the independent therapeutic contribution of HA in the high-risk infected DFU subgroup cannot be extracted from the currently available heterogeneous datasets. A clinical DFU population in which infection status was not reported or stratified can support a general healing signal but not infection resolution or biofilm control. Cell-, PRF- or HYAFF-based complex interventions also do not isolate the contribution of HA. The infection status is explicitly noted in Table 4.

**TABLE 4:** Clinical evidence for HA-containing interventions in diabetic foot ulcers in humans.

Study/reference	Design and sample	Ulcer population	Infection status	HA intervention and comparator	Main outcomes	Principal evidence limitations
(Yao et al., 2025)	Systematic review and meta-analysis	Heterogeneous DFU populations	Mixed/inconsistently stratified	HA-containing dressings or derivatives vs. various comparators	Possible improvement in healing and time to wound closure	Small heterogeneous trials; infection, biofilm, perfusion and background standard care were inadequately stratified
(Lee et al., 2016)	Randomized placebo-controlled; 34 randomized samples	Eligible DFUs	Not reported/not stratified	Pure HA dressing vs. conventional dressing	Increase in the number of cases that reported complete healing and faster healing	Small single-center trial, attrition and limited infection/perfusion data
(You et al., 2014)	Multicenter randomized; 65 patients enrolled	DFUs receiving an autologous construct	Not reported/not stratified	Fibroblast-HA complex vs. nonadherent foam	Improved healing-related outcomes with acceptable short-term safety	Complex cell therapy; the treatment effect cannot be attributed to HA alone; manufacturing and cost limit generalizability
(Caravaggi et al., 2003)	Multicenter randomized; 79 patients enrolled	Plantar or dorsal DFUs	Explicitly not infected	HYAFF-11 autografts vs. paraffin gauze	No significant overall difference in wound closure; possible benefit in the dorsal ulcer subgroup	Not applicable to infected DFUs; older complex technology and subgroup uncertainty
(Uccioli et al., 2011)	Multicenter randomized; 180 patients enrolled	Wagner grade 1–2 DFUs	Not reported/not stratified	Two-step HYAFF autograft vs. nonadherent paraffin gauze	No clear superiority in complete wound closure; modestly shorter mean time to wound closure	Resource-intensive complex intervention; infection-specific contribution cannot be extracted
( Kartika et al., 2021)	Open-label randomized; 30 patients enrolled	Wagner grade 2 DFUs	Not adequately stratified	Advanced PRF plus HA vs. PRF alone vs. saline	Biomarker and granulation-related changes were reported	Very small open-label study; biomarker-focused; HA contribution cannot be separated from that of PRF.
(Vazquez et al., 2003)	Observational cohort; 36 patients enrolled	Neuropathic plantar diabetic foot wounds	Not reported/not stratified	Hyaluronan-containing Hyalofill dressing; no parallel comparator	Seventy-five percent of the wounds were healed within 20 weeks	Uncontrolled study; no infection-, biofilm- or perfusion-specific causal inference

Almost all clinical studies enrolled general DFU, populations and did not prospectively stratify infection, biofilm burden or baseline perfusion. These studies cannot isolate the independent effect of HA, in the high-risk infected DFU, subgroup, and findings from explicit noninfected or heterogeneous DFU, cohorts should not be extrapolated directly to infected DFUs.

Abbreviations: DFU, diabetic foot ulcer; HA, hyaluronic acid; HYAFF, esterified hyaluronan scaffold; PRF, platelet-rich fibrin.

These studies provide preliminary support for the wound-healing potential of HA-containing interventions, but their applicability to infected DFUs remains constrained by limited infection-specific enrollment and incomplete reporting of background standard care.

### 6.4 Material standardization, purification and endotoxin control

Sterilization is part of a product definition rather than a final administrative step. Heat, radiation, filtration or chemical methods can alter HA chain length, crosslinking, mechanical behavior and biological cargo. The corrected Bento et al. (2023) review has been cited for sterilization method selection and reporting; method suitability must still be demonstrated for the specific final formulation, including poststerilization endotoxin and potency testing.

The HA source, molecular weight distribution, purity, degree of substitution, presence of residual reagents, crosslinking, rheology, swelling, degradation, sterilization, storage and batch variability must be reported (Huerta-Ángeles et al., 2018; Faria et al., 2018).

Endotoxin control is particularly important when inflammatory outcomes are central. Residual endotoxin, proteins, nucleic acids or microbial cell wall material from animal-derived or fermented HA can confound cytokine and immune cell readouts (Rodriguez-Marquez et al., 2022; Dong et al., 2016). Every future HA-DFU study should report the full purification workflow, the assay and acceptance threshold for endotoxin, the results for each relevant batch and controls that distinguish contaminant-driven inflammation from intrinsic HA or fragment activity.

### 6.5 Safety, functional trade-offs and temporal programming

Manufacturing feasibility is inseparable from safety. Complex formulations increase the number of critical quality attributes, release specifications and failure modes and may include multiple regulatory categories. Batch release should therefore include identity, potency, residual reagents, sterility/endotoxin presence, and mechanical and release attributes that are linked to the proposed mechanism. Cost and shelf life should be considered before a platform is advanced solely because it performs well in a small animal wound closure model.

Metal ions, cationic antimicrobial agents, photothermal agents, photosensitizers and nitric oxide donors may damage fibroblasts, keratinocytes, endothelial cells or immune cells when dose, energy or release is poorly controlled (Fadeel et al., 2018). Growth factors and vesicles introduce off-target risks of angiogenesis, abnormal granulation, immunological reactions and product variability. Safety assessments must include therapeutic selectivity, degradation products, local retention, systemic exposure and delayed effects.

Formulations combining several distinct therapeutic moieties require substantially more rigorous long-term local and systemic safety monitoring in clinical trials. Cumulative toxicity, unanticipated interactions or delayed off-target effects may not be detected in short-term preclinical studies designed primarily to demonstrate accelerated wound closure. Monitoring should extend beyond initial healing and include recurrent inflammation or infection, sensitization, abnormal vascular or granulation responses, retained material, organ exposure and platform-specific delayed adverse events.

The biological priority also changes over time. Early phases may require debridement, drainage, antimicrobial activity and biofilm control; intermediate phases may require the attenuation of excessive inflammation and oxidative injury; and later phases may favor angiogenesis, ECM reconstruction and re-epithelialization. Programmed, sequential or stage-responsive release is therefore more defensible than simultaneous maximal activation of every function.

### 6.6 Clinical translation, outcomes and a bound role for AI

Protocols should prespecify how repeat debridement, rescue therapy, revascularization, treatment discontinuation, amputation and death are managed in the estimand and analysis (Kahan et al., 2024). Follow-up must extend beyond initial epithelial wound closure to confirm durability and recurrence. Infection- and perfusion-stratified subgroup analyses should be planned prospectively rather than explored after an overall heterogeneous result is obtained. Recent systematic and comparative evidence confirms that recurrence is a distinct endpoint after wound closure (Sun et al., 2024; Lázaro-Martínez et al., 2024). A 14-patient pilot study of plantar cross-linked HA injection provided preliminary evidence of recurrence prevention only and should not be generalized to HA dressings or infected open ulcers (Hughes et al., 2026).

Trials should prospectively define infection severity, microbiology, osteomyelitis, perfusion, wound depth, necrosis and exudate and should standardize or rigorously document debridement, antimicrobial treatment, offloading and vascular care. The outcomes should include complete and sustained healing, time to confirmed wound closure, infection resolution, antimicrobial exposure, repeated debridement, recurrence, minor and major amputation, hospital use, quality of life, cost and long-term safety (Staniszewska et al., 2024; Hopewell et al., 2025; Chan et al., 2025).

AI may serve as a future tool to enable wound measurement, tissue segmentation, phenotype identification, formulation optimization, and treatment response monitoring. Multimodal models may assist in wound measurement, tissue segmentation, phenotyping, formulation optimization or response monitoring, but two-dimensional images alone omit infection, perfusion, depth, loading and background care. Multicenter datasets, external validation and interpretable outputs are needed before AI can support material selection (Silva et al., 2025; Collins et al., 2024).

Table 5 summarizes the minimum domains required to connect material quality, claim-matched mechanisms, clinically relevant models, added benefits, safety and feasibility. The anticipated endpoints in the graphical abstract are targets for future translation and should not be interpreted as outcomes already demonstrated by HA-based materials.

**TABLE 5:** Study design and translational evaluation criteria for HA-based biomaterials intended for the treatment of infected diabetic foot ulcers.

Evaluation domain	Recommended reporting/observations	Suggested endpoints	Translational application	Common weakness (es)	Representative reference(s)
Baseline phenotype	Infection grade, microbiology, biofilm, perfusion, neuropathy, plantar pressure, osteomyelitis, wound depth, necrosis and exudate	IWGDF/IDSA infection grade; SINBAD/WIfI; perfusion measures; wound area/volume	Defines the intended indication and enables clinically interpretable baseline strata	Idealized dorsal wounds or heterogeneous trials without adequate phenotypic data	(Schaper et al., 2024)
Standard care	Debridement and drainage, antimicrobial therapy, offloading, vascular assessment/revascularization and metabolic management	Number/timing of debridements; antibiotic exposure; offloading adherence; revascularization/perfusion improvement	Allows estimation of the added benefit of the material as an adjunct to standard care	Unequal or incompletely reported cointerventions	(Chen, Vilorio, et al., 2024)
Material identity/purity	HA source, molecular weight distribution, purification workflow, endotoxin assay/results/acceptance threshold, protein or nucleic acid contaminants, degree of substitution, residual reagents, sterilization, storage and batch identity	Endotoxin per batch; chemistry; rheology; swelling; degradation; release profile; batch variability	Supports reproducibility, valid immune interpretation, quality control and regulatory translation	Calling a product an ‘HA hydrogel’ without documenting the source, purification protocol, presence of endotoxin or final product properties	(Bento et al., 2023) (Dong et al., 2016)
Component attribution	HA-only, cargo-only, alternative matrix and full composite controls where feasible	Effect size for each component; interaction or factorial analyses	Separates the contribution of the HA matrix from that of active cargo or copolymers	Assigning the efficacy of the complete multicomponent formulation to HA.	(He et al., 2024)
Infection/biofilm	Pathogen spectrum, biofilm maturity, tissue invasion, viable burden, regrowth and recurrence	CFUs; biofilm architecture; infection resolution; reinfection; antimicrobial duration of action	Supports infection-specific antimicrobial or antibiofilm claims	Using planktonic assays or sterile diabetic wounds to support infection treatment claims	(Theodorakopoulos and Armstrong, 2025)
Immune/redox	Functional innate immune cell responses, cytokines, temporal immune phenotypes, redox balance and host cell protection	Phagocytosis; cytokine profile; ROS; SOD; GSH; MDA; Nrf2/HO-1	Tests coordinated the modulation of inflammation/redox while preserving host defense	Relying on M1/M2 markers alone or equating maximal ROS removal with therapeutic benefit	(Clayton et al., 2024)
Vascular/ECM	Vessel maturity, tissue perfusion, oxygenation, matrix organization, MMP/TIMP balance and epithelial continuity	CD31 + alpha-SMA; Doppler/perfusion; oxygenation; MMP/TIMP; collagen I/III; epithelial continuity	Links histological changes to functional vascular and matrix repair	Using VEGF/CD31 staining or wound area closure alone without functional perfusion or tissue quality evidence	(Rai et al., 2023) (Fu et al., 2022)
Clinical outcomes	Complete and sustained healing, infection resolution, recurrence, minor/major amputation, pain, mobility, quality of life and resource use	Time to confirmed wound closure; sustained healing; recurrence; hospital use; patient-reported outcomes; cost	Measures patient-centered effectiveness rather than short-term changes in the wound area alone	Short follow-up, surrogate endpoints and failure to assess recurrence or limb outcomes	(Staniszewska et al., 2024)
Long-term safety	Local cytotoxicity, irritation, sensitization, abnormal granulation/angiogenesis, drainage, retained material, systemic distribution and delayed/cumulative effects	Adverse events through extended follow-up; systemic exposure and organ safety; platform-specific delayed events	Captures cumulative, delayed and multicomponent risks that short-term wound closure studies may miss	Short-term preclinical or clinical studies without adequate local and systemic safety monitoring	(Junqueira et al., 2023)

Claims should be supported by mechanism-specific measurements, tissue-level evidence, clinically relevant endpoints and long-term safety monitoring appropriate for every therapeutic moiety. Trial estimands and intercurrent events, including repeat debridement, rescue therapy, revascularization, treatment discontinuation, amputation and death, should be prespecified in the main trial methods.

Abbreviations: CFU, colony-forming unit; DFU, diabetic foot ulcer; GSH, glutathione; HA, hyaluronic acid; HO-1, heme oxygenase-1; IWGDF, international working group on the diabetic foot; MDA, malondialdehyde; MMP, matrix metalloproteinase; Nrf2, nuclear factor erythroid 2-related factor 2; ROS, reactive oxygen species; SINBAD, site, Ischemia, Neuropathy, Bacterial infection, Area and Depth; SOD, superoxide dismutase; TIMP, tissue inhibitor of metalloproteinase; WIfI, wound, Ischemia and foot Infection.

## 7 Conclusion

Infected DFUs are sustained by interactions among microbes and inflammatory, oxidative, vascular, mechanical and matrix abnormalities. HA-based tissue-engineered materials may provide a hydrated matrix, tunable chemistry, local retention and controlled presentation of therapeutic cargo, but they must remain adjuncts to debridement, infection management, offloading and vascular optimization.

The evidence base is uneven. Only a limited subset of studies have used explicit infected diabetic wound models; much of the evidence concerning antioxidant, angiogenic, immunomodulatory and ECM effects is derived from noninfected diabetic wounds or *in vitro* systems. Published clinical studies have focused mainly on general DFU populations without adequate infection, biofilm or perfusion stratification. Direct effectiveness in high-risk infected DFU patients therefore remains unestablished.

Translation should distinguish the function of HA from that of the active cargo; use component deconvolution controls; match models to the therapeutic claim; document the source, molecular weight, purification and endotoxin burden; and evaluate long-term local and systemic safety. Phenotype-informed and stage-responsive designs are promising research hypotheses, not current clinical algorithms. Progress will be measured by infection resolution, durable healing, recurrence, limb and patient-centered outcomes rather than accelerated short-term wound closure alone.
